# General Practitioners’ Barriers to Prescribe Physical Activity: The Dark Side of the Cluster Effects on the Physical Activity of Their Type 2 Diabetes Patients

**DOI:** 10.1371/journal.pone.0140429

**Published:** 2015-10-15

**Authors:** Charlotte Lanhers, Martine Duclos, Aline Guttmann, Emmanuel Coudeyre, Bruno Pereira, Lemlih Ouchchane

**Affiliations:** 1 Department of Sport Medicine and Functional Explorations, Clermont-Ferrand University Hospital (CHU), BP 68, 63001, Clermont-Ferrand, Cedex 1, France; 2 Laboratory of Human Nutrition, INRA UMR 1019, Clermont-Ferrand, France; 3 Clermont-Ferrand University Hospital, Department of Public Health, Biostatistics Unit, Clermont-Ferrand, France; 4 Laboratory of Image Sciences for Interventional Techniques, UMR CNRS UdA 6284 ISIT, Auvergne University, Clermont-Ferrand, F-63001, France; 5 Clermont University, University of Auvergne, Clermont-Ferrand, France; 6 Clermont-Ferrand University Hospital, Department of Physical Medicine and Rehabilitation, Clermont-Ferrand, France; 7 Clermont-Ferrand University Hospital, Innovation and Clinical Research, Clermont-Ferrand, France; University of Catanzaro Magna Graecia, ITALY

## Abstract

**Aims/hypothesis:**

To describe barriers to physical activity (PA) in type 2 diabetes patients and their general practitioners (GPs), looking for practitioner’s influence on PA practice of their patients.

**Methods:**

We conducted a cross-sectional study on GPs (n = 48) and their type 2 diabetes patients (n = 369) measuring respectively barriers to prescribe and practice PA using a self-assessment questionnaire: barriers to physical activity in diabetes (BAPAD). Statistical analysis was performed accounting hierarchical data structure. Similar practitioner’s patients were considered a cluster sharing common patterns.

**Results:**

The higher the patient’s BAPAD score, the higher the barriers to PA, the higher the risk to declare practicing no PA (p<0.001), low frequency and low duration of PA (p<0.001). A high patient’s BAPAD score was also associated with a higher risk to have HbA_1c_ ≥7% (53 mmol/mol) (p = 0.001). The intra-class correlation coefficient between type 2 diabetes patients and GPs was 34%, indicating a high cluster effect. A high GP’s BAPAD score, regarding the PA prescription, is predictive of a high BAPAD score with their patients, regarding their practice (p = 0.03).

**Conclusion/interpretation:**

Type 2 diabetes patients with lower BAPAD score, thus lower barriers to physical activity, have a higher PA level and a better glycemic control. An important and deleterious cluster effect between GPs and their patients is demonstrated: the higher the GP’s BAPAD score, the higher the type 2 diabetes patients’ BAPAD score. This important cluster effect might designate GPs as a relevant lever for future interventions regarding patient’s education towards PA and type 2 diabetes management.

## Introduction

Physical activity (PA) is a cornerstone in type 2 diabetes management, significantly improving glycemic control, lowering HbA_1c_ by an average of 0.6–0.8% [1,2]. This is clinically relevant since HbA_1c_ reduction is associated with improved morbidity and mortality outcomes [3].

The American Diabetes Association emphasizes the benefits of regular PA in the treatment of type 2 diabetes and advises to engage in moderate-intensity PA for at least 30 min on most days of the week [4]. However, despite the promotion of an active lifestyle from multilevel agencies, it is apparent that too few type 2 diabetes patients follow these recommendations. Actually, adults with diabetes are less likely to engage in regular PA than the general adult population [5] and only 23% of older adults with type 2 diabetes report more than 60 min of weekly PA in the US [6]. Therefore, factors that impede PA in type 2 diabetes need to be identified in order to guide future interventions.

To meet the challenges facing type 2 diabetes, interventions need to acknowledge constraints on behavioral changes and to identify effective strategies to reverse behavioral trends towards physical inactivity [7]. Representations and beliefs with type 2 diabetes patients and their general practitioners (GP) regarding PA practice might likely be wrong, so deleterious. Assuming that GPs’ promotion and prescription of PA influence actual practice in type 2 diabetes patients, GPs’ beliefs towards PA in these patients might be a key issue.

In 2006, Dube et al. [8] developed a questionnaire (Barriers to Physical activity in Diabetes: BAPAD) that measured perceived barriers to practice regular PA for diabetes patients in order to identify potential targets for future interventions.

We aimed to investigate the possible link between PA in type 2 diabetes patients and GP’s attitude regarding PA promotion. To carry out such an investigation testing for this possible influence of GP’s on their patients, we performed a survey over a whole French region and simultaneously measured barriers to PA practice in type 2 diabetes patients using the original version of BAPAD questionnaire and the GPs’ reluctance in prescribing PA for such patients using an adapted version of BAPAD questionnaire.

## Methods

### Study design

We conducted a cross-sectional study on GPs and their type 2 diabetes patients in the Auvergne region of France. All the private GPs (n = 970) practicing in this region were invited to participate in our survey. They were mailed a packet that included a letter describing the study, an acceptance/refusal form, and a postage-paid return envelope. A phone-call reminder was made for non-respondents 3 weeks after the packet was mailed. GP’s written consent was actually the first condition to participate in the survey and start patient’s proposal to enter the survey. A medical representative met each physician who agreed to participate 5–6 weeks after the initial mailing, and delivered a form for the physician (GP’s form) and a set of additional forms intended for their type 2 diabetes patients (patients forms). Each GP was asked to enroll up to 10 patients with type 2 diabetes. The only exclusion criterion was treatment with insulin (S1 Fig). The instructions were first to complete the GP’s form and then to start including patients, allowing them to complete the form on their own. Each physician was assigned a number to return with the questionnaire. The number assigned to each physician was attached to a number assigned to each of his/her patient. This was only done to relate each patient to his physician while keeping the data file anonymous. About 4 weeks later, the same medical representative collected the forms during a meeting with the GP. The study was conducted, by the University Hospital of Clermont-Ferrand and was approved by the appropriate IRBs (CCPPRB d’Auvergne for any clinical study and CPP Sud-Est VI specifically for observational study) in accordance with the protocol of Helsinki. Our local IRB approved the whole content of the protocol of our survey, including all participants’ recruitment (GPs and patients). All the information used and analyzed in our survey was obtained exclusively from self-administered forms and our study was completely anonymous regarding both GPs and patients whose linkage was carried-out through numbers assigned to patients which included the number of their corresponding GP.

### Forms and questionnaires

Patient data were collected during a visit to their GPs. The first part of the patient’s self-administered form asked for demographic data history, and if they knew the last value of their HbA_1c_, and if so, its value. This first part of the questionnaire also asked the patient both the frequency, i.e. number of at least 30 min long sessions per week (categorized as “1–2 times/week”, “3–4” or “everyday”) and the duration of PA per week (categorized as “under 2h”, “2-3h” or “over 3h”). “Regular PA” was defined as “2h or more per week at least 3 times per week”. The strata “1 to 2 times per week” (for frequency) and “under 2h” (for duration) were considered the reference categories to display the results. A second section of the questionnaire dealt with their GP’s attitude regarding behavioral modifications. Type 2 diabetes patients were asked to rate the relative importance of the three following therapeutic measures to manage their disease: glucose-lowering medication, regular PA and quitting smoking. A third section of the questionnaire assessed the type 2 diabetes patient’s barriers to PA using the original version of the BAPAD questionnaire.

The GP’s form was based on a similar self-administered form, and also included three parts. The first part sought information concerning demographic data, professional data and personal PA. The second part inquired about GPs’ medical care for type 2 diabetes, with particular emphasis on promotion or education on PA for the management of type 2 diabetes. The third part assessed the GPs’ barriers to promote or prescribe PA to their type 2 diabetes patients through a slightly adapted version of the BAPAD questionnaire.

The original BAPAD questionnaire measures the perceived barriers towards undertaking regular PA by type 2 diabetics using 11 items [8]. Patients are asked to rate, using a seven-level rating scale, the likelihood that each of the 11 items would keep them from practicing regular PA during the next 6 months (Table 1). We slightly adapted the phrasing to evaluate the GPs’ barriers regarding the prescription of PA while using the same seven-level rating scale to indicate the likelihood that each of the same 11 items would keep them from prescribing regular PA during the next 6 months to their type 2 diabetes patients.

**Table 1:** The 11 items of the BAPAD with percentages and mean scores (±SE) per item for both GPs and their type 2 diabetes patients (GPs/Patients).

BAPAD Items, GPs (A)/type 2 diabetes patients (B). Percentages for each rating level and GPs’/type 2 diabetes patients’ BAPAD score Mean ±SE	Seven-level rating scale							
	1	2	3	4	5	6	7	Mean ±SE
**1-Loss of control for diabetes**	71/44	15/17	4/9	6/16	2/6	0/5	2/4	1.63±0.2/2.51±0.1
**2-The risk of hypoglycemia**	35/30	31/15	13/13	8/15	4/12	4/7	4/7	2.44±0.2/3.14±0.1
**3-The fear of being tired**	60/25	25/12	2/11	8/13	2/15	0/17	2/7	1.75±0.2/3.59±0.1
**4-The fear of hurting**	60/41	19/17	10/9	6/12	4/8	0/8	0/5	1.75±0.2/2.76±0.1
**themselves**								
**5-The fear of suffering a heart**	25/37	19/12	19/9	21/16	6/10	8/11	2/6	2.98±0.2/3.06±0.1
**attack**								
**6-A low fitness level**	23/21	23/10	19/12	10/16	13/13	10/17	2/11	3.06±0.3/3.85±0.1
**7-The fact that they have**	77/46	17/14	4/10	0/15	0/7	2/5	0/3	1.35±0.1/2.49±0.1
**diabetes**								
**8-The risk of hyperglycaemia**	85/55	13/17	0/8	0/11	0/3	2/2	0/2	1.23±0.1/2.07±0.1
**9- Physical health status**	6/29	6/16	13/5	29/14	21/11	15/9	10/16	4.38±0.2/3.53±0.1
**10-Weather conditions**	27/23	17/10	25/11	17/12	6/16	4/17	4/11	2.88±0.2/3.83±0.1
**11-Their work schedule**	33/57	15/6	17/7	15/9	10/5	6/5	4/11	2.90±0.3/2.56±0.1
**Total**	46/37	18/13	11/10	11/14	6/10	5/9	3/8	2.39±0.1/3.04±0.1

A: For GPs: “Indicate the likelihood that each of the 11 items would keep you from prescribing regular physical activity during the next 6 months to your type 2 diabetes patients.” B: For type 2 diabetes patients: “Indicate the likelihood that each of the 11 items would keep you from practicing regular physical activity during the next 6 months.” (Ranging from 1 = very unlikely, 2 = unlikely, 3 = rather unlikely, 4 = uncertain, 5 = rather likely, 6 = likely, 7 = very likely).

### Statistical analyses

Continuous variables were displayed as mean±standard error (mean±SE). Categorical and ordinal variables were described using frequencies and percentages. For each item of the BAPAD questionnaire, we displayed both the distribution and summary statistics (mean±SE). The BAPAD score was defined as the mean of the 11 BAPAD items.

Because we used the BAPAD scale for type 2 diabetes patients and their GPs, we assessed the internal consistency of the BAPAD scale using the alpha Cronbach’s coefficient. This coefficient was of 0.85 in Dube et al. who used the BAPAD scale for type 1 diabetes patients [8].

By comparing the Cronbach’s coefficients, we aimed to check that the reliability of the BAPAD scale for type 2 diabetes patients and their GPs was at least as satisfactory as for type 1 diabetes patients.

Regarding GPs’ characteristics and their BAPAD score, relationships between categorical variables were assessed using Fisher’s exact test, relationships between continuous variables were assessed using Spearman’s correlation coefficient test, and relationships between categorical and continuous variables were assessed using, as appropriate, either a Wilcoxon test or a one-way Kruskal-Wallis ANOVA.To further analyze GPs’ influence regarding PA of type 2 diabetes patients, we used random-effect models accounting for hierarchical data structure (since patients with the same GP are considered a cluster). Intra-cluster correlation coefficient (ICC) [9] was computed.

Subsequent analyses all accounted for cluster effect and consisted in modeling patients’ BAPAD score as a continuous response variable. A first model included only the patient’s characteristics (as explanatory variables), such as the disease history or the HbA_1c_ level, and then a larger one extended explanatory variables to GPs’ characteristics, including GPs’ BAPAD score. Explanatory variables selections were performed through automated stepwise procedures. Less than 5% of the data were incomplete and a sensitivity analysis was performed to confirm the robustness of our results. All statistical analyses were performed using Stata software (StataCorp, College Station, TX, US) with a two-tailed type-I error set to 0.05.

## Results

Among the 970 GPs asked to participate, 658 (68%) responded. Among these, 574 refused to participate and 84 (13%) agreed. Among these 84 GPs, 36 (43%) did not include patients or did not provide a complete response to the form, and so were excluded from the analysis. This left a total of 48 GPs, and their 369 patients, who were included in our study (S1 Fig).

GPs who answered the survey were aged 48.9±1.2, and included 29 males (60.4%) and 19 females (39.6%) (Table 2). The mean number of patients included per GP was 7.7±0.9.

**Table 2:** GPs’ BAPAD score regarding GPs’ characteristics (A), type 2 diabetes patients’ BAPAD score regarding patients’ characteristics (B).

**A : GPs’ characteristics**		**N**	**(%)**	**GP’s BAPAD**	***p*-value**
Department	Allier (03)	4	(8.3)	2.16±0.21	0.70
Department	Cantal (15)	3	(6.3)	2.30±0.03	0.70
Department	Haute-Loire (43)	8	(16.7)	2.09±0.31	0.70
Department	Puy-de-Dôme (63)	33	(68.8)	2.50±0.17	0.70
GP’s gender	Female	19	(39.6)	2.51±0.23	0.82
GP’s gender	Male	29	(60.4)	2.32±0.15	0.82
Location	Rural	15	(31.3)	2.45±0.29	0.90
Location	Urban	21	(43.8)	2.41±0.19	0.90
Location	Both	12	(25.0)	2.29±0.16	0.90
Physical activity	None	4	(8.3)	2.23±0.41	0.84
Physical activity	Occasionally	24	(50.0)	2.38±0.19	0.84
Physical activity	Regularly	20	(41.7)	2.44±0.20	0.84
Time spent prescribing exercise	<10 min	40	(40)	2.46±0.14	0.43
Time spent prescribing exercise	>10 min	8	(8)	2.08±0.34	0.43
Medical training on DT2	No	9	(18.8)	2.34±0.13	0.97
Medical training on DT2	Yes	39	(81.3)	2.41±0.15	0.97
Medical training on activity	No	23	(47.9)	2.65±0.17	0.05
Medical training on activity	Yes	25	(52.1)	2.16±0.17	0.05
Medical training on nutrition	No	14	(29.2)	2.38±0.13	0.78
Medical training on nutrition	Yes	34	(70.8)	2.40±0.17	0.78
Medical training on medication	No	13	(27.1)	2.22±0.14	0.48
Medical training on medication	Yes	35	(72.9)	2.32±0.17	0.48
Wanting new medical training	No	26	(54.2)	2.26±0.17	0.18
Wanting new medical training	Yes	22	(45.8)	2.55±0.18	0.18
B : Type 2 diabetes patients characteristics		**N**	**(%)**	**Patient’s BAPAD**	***p*-value**
				**score Mean±SE**	
Patient gender	Female	151	(40.9)	3.17±0.11	0.14
Patient gender	Male	218	(59.1)	2.95±0.09	0.14
Hba1c value known	No	125	(33.9)	3.29±0.12	0.007
Hba1c value known	Yes	244	(66.1)	2.90±0.08	0.007
Regular physical activity	No	192	(52.0)	3.40±0.09	<0.0001
Regular physical activity	Yes	177	(48.0)	2.64±0.09	<0.0001
Cycling	No	328	(88.9)	3.09±0.07	0.02
Cycling	Yes	41	(11.1)	2.59±0.17	0.02
Swimming	No	355	(96.2)	3.05±0.07	0.16
Swimming	Yes	14	(3.8)	2.55±0.25	0.16
Walking	No	182	(49.3)	3.33±0.10	<0.0001
Walking	Yes	187	(50.7)	2.75±0.09	<0.0001
Physical activity frequency (times per week)	1–2	200	(54.2)	3.38±0.09	<0.0001
Physical activity frequency (times per week)	3–4	70	(19.0)	2.78±0.14	<0.0001
Physical activity frequency (times per week)	>4	99	(26.8)	2.52±0.11	<0.0001
Physical activity duration (h per week)	<2	183	(49.6)	3.39±0.10	<0.0001
Physical activity duration (h per week)	2–3	76	(20.6)	2.98±0.14	<0.0001
Physical activity duration (h per week)	>3	110	(29.8)	2.49±0.11	<0.0001
Asked to stop smoking	No	230	(62.3)	2.91±0.08	0.013
Asked to stop smoking	Yes	139	(37.7)	3.24±011	0.013
Asked to lose weight	No	82	(22.2)	2.45±0.13	<0.0001
Asked to lose weight	Yes	287	(77.8)	3.20±0.08	<0.0001
Asked to practice activity	No	62	(16.8)	2.79±0.18	0.04
Asked to practice activity	Yes	307	(83.2)	3.08±0.07	0.04

Regarding their personal PA, most declared occasional (n = 24, 50%) to regular PA (n = 20, 41.7%) at least three times per week, and only four (8.3%) declared no PA at all (Table 2). All but one GP reported questioning their type 2 diabetes patients about their level of PA.

The GPs’ prescriptions showed that in first intention 43 GPs (90%) prescribed walking, 39 (81%) prescribed stopping smoking, 47 (98%) prescribed losing weight and 45 (94%) prescribed PA.

Within a total rating of 10 points, GPs ranged their priority among three measures for managing diabetes, resulting in a mean score of 6.3±0.3 points for lifestyle measures (exercise and nutrition), 2.8±0.3 for a mono-therapy (one glucose-lowering medication) and 0.9±0.2 for a bi-therapy. All the GPs declared allotting time during their consultation to prescribing PA, however, most (n = 40, 83.3%) spent less than 10 min (Table 2).

A huge majority of GPs (n = 39, 81.3%) had received at least one medical training session on the management of type 2 diabetes in the last 3 years, which had focused on PA, nutrition or medication. Only a minority (n = 22, 45.8%) wanted a new medical training on PA.

No significant association was found between any of the demographic or professional characteristics of the GPs and their BAPAD scores. Similarly, we did not find any association between GPs’ BAPAD score and GPs’ level of PA, prescription to stop smoking, to lose weight, to practice PA or walking. Regarding the remaining GPs’ characteristics, a slight trend was found for medical training, with a lower BAPAD score for GPs who had previous training on PA (p = 0.06).

A total of 369 patients aged 64.9±0.5 entered the survey, including 218 males (59%) and 151 females (41%). Diabetes had been discovered 9.7±7.6 years prior to the study. In addition, 244 (66%) knew their last HbA_1c_ value, which was 7.27±0.08% (56 mmol/mol). When asked whether they had regular PA (at least 30 min on each occasion), 177 (48%) answered positively. However, only 169 (45.8%) declared practicing PA at least three times a week and among them, 99 (26.8%) had daily PA. Regarding duration and frequency, 146 out of 369 (36.9%) fulfilled the definition of regular PA (Table 2).

Most patients (n = 307, 83.2%) had been asked by their GPs to practice PA since the discovery of their type 2 diabetes (Table 2). However, unlike their GPs, type 2 diabetes patients gave the greatest priority to medication to manage their diabetes, with a mean value of 6.3±0.1 within a total rating of 10 points. The role of PA was given in second position, with a mean of 2.9±0.1.

The internal consistency of the BAPAD scale assessed by the alpha Cronbach’s coefficient was of 0.87 regarding patient’s questionnaire, which indicates that the reliability of the barriers scale is very satisfactory, at least as satisfactory as in Dube et al. who found a coefficient of 0.85 for BAPAD questionnaire in type 1 diabetes [8]. The alpha Cronbach’s coefficient for GPs questionnaire was 0.83 which appeared also acceptable according to the literature [10].

The GPs’ and patients’ BAPAD scores are respectively described with mean±SE (Table 1). Analyzing GPs’ and patients’ BAPAD, we found that the main barriers held in common between patients and their practitioners were “the fear of suffering a heart attack” (2.9±0.2/3.1±0.1, “their physical health status” (4.4±0.2/3.5±0.1), and “a low fitness level” (3.1±0.3/3.9±0.1).

The clustering effect evaluated by ICC between type 2 diabetes patients and GPs was 34%, which is considered to be a high value [9]. Using mixed model, type 2 diabetes patient’s BAPAD score was linked with all patient’s characteristics including gender (Table 3). A high BAPAD score reflecting a high barrier to PA was significantly and positively correlated with duration of diabetes (> 8 years) (p = 0.002). The patient’s BAPAD score was significantly higher with patients whose HbA_1c_ was known (p = 0.001), and among these patients (n = 244) with those with HbA_1c_ ≥ 7% (53 mmol/mol) (p = 0.001).

**Table 3:** Patient’s BAPAD according to patient’s characteristics (univariate mixed model).

		Patient’s BAPAD	*p-*value
		Mean ± SE	
Age	<65	2.99 ± 0.07	**0.02**
Age	≥65	3.08 ± 0.06	**0.02**
Gender	Male	2.95 ± 0.09	**0.02**
Gender	Female	3.17 ± 0.11	**0.02**
Duration diabetes (year)	≤4	2.79 ± 0.06	**.**
Duration diabetes (year)	4–8	3.02 ± 0.07	**0.17**
Duration diabetes (year)	>8	3.19 ± 0.07	**0.002**
HbA_1c_ value known	No	3.29 ± 0.12	**0.001**
HbA_1c_ value known	Yes	2.90 ± 0.08	**0.001**
HbA_1c_ value	<7(53 mmol/mol)	2.62 ± 0.06	**0.001**
HbA_1c_ value	≥7(53 mmol/mol)	3.15 ± 0.07	**0.001**
Regular physical activity	No	3.40 ± 0.09	**<0.001**
Regular physical activity	Yes	2.64 ± 0.09	**<0.001**
Physical activity frequency (times /week)	1–2	3.38 ± 0.09	**.**
Physical activity frequency (times /week)	3–4	2.78 ± 0.14	**<0.001**
Physical activity frequency (times /week)	>4	2.52 ± 0.11	**<0.001**
Physical activity duration (h /week)	<2	3.39 ± 0.18	**.**
Physical activity duration (h /week)	2–3	2.98 ± 0.14	**0.004**
Physical activity duration (h /week)	>3	2.49 ± 0.11	**<0.001**
Asked to stop smoking	No	2.91 ± 0.08	**0.83**
Asked to stop smoking	Yes	3.24 ± 0.11	**0.83**
Asked to lose weight	No	2.45 ± 0.13	**0.02**
Asked to lose weight	Yes	3.20 ± 0.08	**0.02**
Asked to practice physical activity	No	2.79 ± 0.18	**0.38**
Asked to practice physical activity	Yes	3.08 ± 0.07	**0.38**
Importance to take medics	ES	0.12 [0.07, 0.18]	**<0.001**
Importance to practice physical activity	ES	-0.14 [-0.20, -0.08]	**<0.001**
Importance to stop smoking	ES	-0.03 [-0.12, 0.05]	**0.47**

Each regression coefficient is interpreted as the effect size (ES).

BAPAD score was significantly higher with patients who declared having no PA (p<0.001), having a low frequency and low duration of PA (p<0.001), and with patients asked to lose weight (p = 0.02). Although not significant, we noticed similar trends regarding both patients asked to practice PA and to stop smoking, (3.08 ± 0.07 (p = 0.38) and 3.24 ± 0.11 (p = 0.83), respectively). Finally, the higher the priority given by patients to medication, the higher their BAPAD score; the higher the priority given to PA, the lower the BAPAD score (Table 3).

Few GPs’ characteristics were significantly linked with the patients’ BAPAD. Among those, the only demographic characteristic was gender: patient whose GP was male had higher BAPAD score (p = 0.01). The higher the GPs’ BAPAD, the higher the patients’ BAPAD score (p = 0.03) (Table 4). Patients’ BAPAD score was not linked with practitioner specialty, GPs’ physical activity, medical training or their individual type 2 diabetes management.

**Table 4:** Patient’s BAPAD according to general practitioner’s characteristics (univariate mixed model).

		Patients’ BAPAD	*p-*value
		Mean ± SE	
Age	<50	2.74 ± 0.06	**0.10**
Age	≥50	3.21 ± 0.07	**0.10**
Gender	H	3.24 ± 0.07	**0.008**
Gender	F	2.58 ± 0.05	**0.008**
Location	Rural	2.85 ± 0.07	**.**
Location	Both	2.89 ± 0.06	**0.64**
Location	Urban	3.20 ± 0.07	**0.48**
Specialty medicines	Endoc	3.09 ± 0.07	**.**
Specialty medicines	Nut	2.44 ± 0.06	**0.55**
Specialty medicines	GPs	2.44 ± 0.06	**0.40**
Physical activity	None	3.59 ± 0.06	**.**
Physical activity	Occasionally	3.14 ± 0.07	**0.14**
Physical activity	Regularly	2.83 ± 0.07	**0.13**
Asking patient if practice PA	No	1.91 ± 0.03	**0.19**
Asking patient if practice PA	Yes	3.10 ± 0.07	**0.19**
Medical training on type 2 diabetes	No	2.60 ± 0.06	**0.46**
Medical training on type 2 diabetes	Yes	3.16 ± 0.07	**0.19**
Wanting new medical training on physical activity	No	3.01 ± 0.07	**0.91**
Wanting new medical training on physical activity	Yes	3.06 ± 0.06	**0.91**
Medical training on activity in type 2 diabetes	No	3.03 ± 0.06	**0.78**
Medical training on activity in type 2 diabetes	Yes	3.04 ± 0.07	**0.78**
Medical training on nutrition in type 2 diabetes	No	2.73 ± 0.06	**0.42**
Medical training on nutrition in type 2 diabetes	Yes	3.18 ± 0.07	**0.42**
Medical training on medication in type 2 diabetes	No	2.65 ± 0.06	**0.41**
Medical training on medication in type 2 diabetes	Yes	3.20 ± 0.07	**0.41**
Time for PA prescription	<10 Min	3.10 ± 0.06	**0.43**
Time for PA prescription	>10 Min	2.70 ± 0.08	**0.43**
Importance for dietary rules	ES	-0.06 [-0.18, 0.04]	**0.22**
Importance for Monotherapy	ES	0.10 [-0.04, 0.25]	**0.18**
Importance for Bitherapy	ES	0.03 [-0.14, 0.20]	**0.72**
GPs’ BAPAD	ES	0.03 [0.01–0.05]	**0.03**

Each regression coefficient is interpreted as the effect size (ES).

In multivariate analysis (S2 Fig), factors significantly and independently associated with patient’s BAPAD were GPs gender (higher BAPAD score for male), HbA_1c_ level (higher BAPAD score if HbA_1c_ ≥ 7% (53 mmol/mol)) and physical activity (lower BAPAD score in patients managing to practice sport frequently). Compared with the reference frequency which was “1 to 2 times per week”, the more frequent the sport practice (3 and 4 per week, every day), the lower the patients BAPAD’s score. Patients asked by their GPs to lose weight exhibited a propensity for a higher BAPAD score. Finally, the higher the GPs’ BAPAD score, the higher the patients’ BAPAD score.

## Discussion

The originality of this study lies in the planned matching between the GPs and their corresponding patients. It enabled refined analyses of the link between patients’ behavior and their GPs by accounting for hierarchical data structure through mixed effects linear model with patients from the same GP considered a cluster. In primary health care settings, cluster sampling is often required when subjects are to be recruited from several practices or practitioners. [11]. Using this method, we confirmed that the higher the GPs’ BAPAD score, the higher the type 2 diabetes patient’s BAPAD score.

In our study, the cluster effect, i.e. the link between GPs and their patients as measured through ICC, was 34%. Developed at the university of Aberdeen, an international database gathering 210 studies includes such ICCs [12] dealing with both GPs and specialists among management of various diseases. However, there is little information available concerning GPs and patients with diabetes, and actually, none of these studies emphasizes ICC for GPs and type 2 diabetes patients. ICCs (for GP clustering) over 20% were unusual (10% of studies). The highest ICC referenced was 28%, emphasizing a strong link between GPs and their patients. The high ICC (34%) we found in our study demonstrated a high cluster effect between type 2 diabetes patients and their GPs, which, to our knowledge, has never been demonstrated.

PA is a central component of type 2 diabetes management. Improvements due to PA in type 2 diabetes include increased insulin sensitivity and responsiveness along with a positive effect on lipids, blood pressure, cardiovascular events, mortality and quality of life [13]. A planned epidemiological study in type 2 diabetes patients showed that a 1% increase of HbA_1c_ level is associated with an increase in the risk of diabetic micro and macroangiopathy [14]. Our results show higher BAPAD scores in patients with inactive lifestyle and also in patients with unbalanced glycemic level. Together with the aforementioned studies’ results [4, 12], it is suggested that high BAPAD scores might consequently reflect a high risk of serious health problems and morbidities associated with type 2 diabetes. However, although PA is presented as a cornerstone for type 2 diabetes patients, it is often difficult to incorporate regular PA into daily lives [11]. Consistent with previous studies [5], our study showed that most adults with type 2 diabetes do not engage regularly in PA as 54% report no regular PA and less than 27% declared having daily PA.

Regarding the main purpose of our study, we found that both practice and representation of PA were reversely associated with BAPAD score of type 2 diabetes patients. Perceived barriers are important in the general field of health behavior and more particularly in practicing PA [15].

Since the main source of information for patients about their diabetes is their GPs, it appeared essential to incorporate not only the characteristics of patients but also the characteristics of their GPs into our analysis seeking explanatory variables of patient’s BAPAD score. According to the Health-Belief Model, perceived barriers decrease the likelihood of engaging in behavior that might otherwise reduce disease risks, enhance health and help control the signs and symptoms once a condition is established [16]. Paradoxically, most people cite their GPs as their primary source of information regarding healthy lifestyle decisions and yet few studies have evaluated the influence of the counselling practice of GPs on the perceived barriers of type 2 diabetes patients towards the practice of PA. Moreover, the possible transmission of barriers to PA from GPs to their type 2 diabetes patients had not been yet assessed.

Thus, identification and improvement of GPs’ barriers to promote PA seem important steps to enhance the PA of type 2 diabetes patients. Actually, it has been shown that endorsement of an active lifestyle is more credible coming from a professional who is physically active [17]. However, in our study, we have not found such a link between patient’s BAPAD score and level of physical activity of GPs. The investigation of general practitioners’ characteristics showed higher BAPAD score in patients whose GP was a man. A possible explanation might be that male GPs are less empathic and take less time for patient education, especially to encourage type 2 diabetes patients to practice PA [18]. Several studies have examined gender differences in preventive services counselling for patients visiting primary care clinics and have found contradictory results. Tabenkin et al [19] showed that, compared with male physicians, female provide more counseling for a variety of cardiovascular risk factors, including diabetes, whereas Kim et al reported that patients of female physicians received similar quality of care compared with patients of male physicians [20].

The main limitation of this study is the possibility of selection bias due to a low overall response rate from GPs (<10%). Such a response rate is quite common according to the literature [21,22]. Our responders probably are GPs more concerned with type 2 diabetes education, and particularly with PA. On the contrary, our sample of GPs may also held incorrect beliefs and not fully understand the benefits of engaging in healthy behaviors, such as PA. Therefore, it seems difficult to know how the low response rate is affecting our results, but one can easily hypothesize that GPs’ barriers towards prescription of PA might have been even higher with physicians who did not answer the survey.

The strength of this study is that it is the first one to assess GPs’ attitudes, knowledge and practical approaches regarding PA in type 2 diabetes and to seek for possible influence on their type 2 diabetes patients.

In our view, it is important to measure GPs’ barriers in promoting PA since they may hamper the message of delivering health-related benefits. Indeed, while examining the nature of perceived barriers to PA in both type 2 diabetes patients and their GPs, we found common barriers emerging with a high score, which is consistent with the hypothesis that the beliefs of GPs might influence the beliefs of their patients. Furthermore, these common barriers are inconsistent with evidence-based medicine, since it has been repeatedly shown that appropriate PA is safe, effective and important for people with disabilities [23]. Thus, identifying and acting on GPs’ barriers might act as a lever to increase type 2 diabetes PA.

The research literature proposes a number of strategies that may help with overcoming the challenge and barriers faced by many type 2 diabetes patients. One particular approach highlighted by our study is the need for GPs to be informed about the effects of PA. It is imperative that physicians who work with type 2 diabetes patients acknowledge not only their patients’ barriers, but also their own, in an attempt to respond to their patients’ specific needs and to deliver a message that encourages participation. Male general practitioners should be particularly targeted regarding patient education.

In conclusion, we have shown an association between GPs’ barriers in promoting PA and type 2 diabetes patients’ barriers in practicing regular PA. The strong cluster effect was not just due to the classical patient based effect. The BAPAD result on diabetic type 2 patients is very dependent from the GPs’ good practice beyond all the characteristics of GPs. These findings suggest that modifiable variables influence type 2 diabetes patients’ response to PA. To meet these challenges, interventions that intend to increase PA should be multifactorial and multidirectional, i.e. directed towards patients and their physicians. This management should be personalized and adapted for patients and their GPs. GPs and patients would benefit from a network of professionals specialized in PA for patients with diabetes. Thus, GPs could prescribe and patients could practice PA safely.

## Supporting Information

S1 Fig Flowchart of study participants (GPs and patients). (TIF)

S2 Fig Multivariate mixed modeling of patient’s BAPAD with patient’s and general practitioner’s characteristics (regression coefficients and 95% CI). (TIF)

S1 File BAPAD_DATA. (PDF)

## Abbreviations

BAPAD: Barriers to Physical activity in Diabetes
PA: Physical activity
GP: General Practitioner
ICC: Intra-cluster correlation coefficient
