# Supplementary figures and images for: General Practitioners’ Barriers to Prescribe Physical Activity: The Dark Side of the Cluster Effects on the Physical Activity of Their Type 2 Diabetes Patients
Source: PLoS One. 2015 Oct 15;10(10):e0140429. doi: 10.1371/journal.pone.0140429 (PMC4607360; doi:10.1371/journal.pone.0140429)

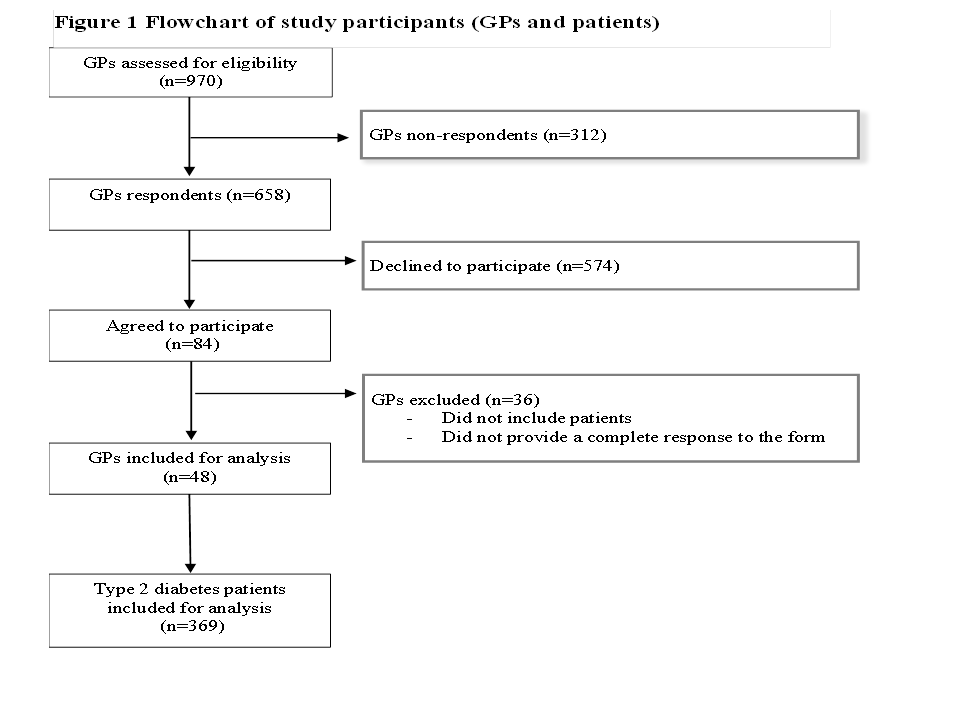

Supplement: S1 Fig — (TIF) [file pone.0140429.s001.tif]

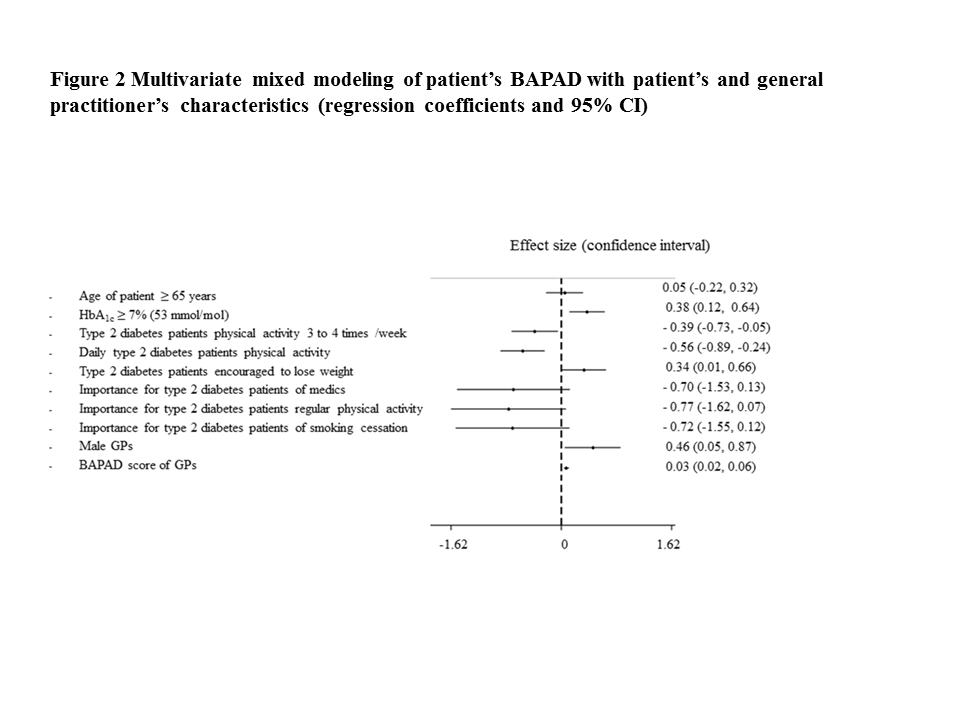

Supplement: S2 Fig — (TIF) [file pone.0140429.s002.tif]
