# Supplementary material for: General Practitioners’ Barriers to Prescribe Physical Activity: The Dark Side of the Cluster Effects on the Physical Activity of Their Type 2 Diabetes Patients
Source: PLoS One. 2015 Oct 15;10(10):e0140429. doi: 10.1371/journal.pone.0140429 (PMC4607360; doi:10.1371/journal.pone.0140429)
Supplement: S1 File — (PDF) [file pone.0140429.s003.pdf]

medecin	nb_patients	datamedecins	depart_med	male_med	age_med	milieu_med	type_med
27	4	1	15	0	36	2	1
27	4	1	15	0	36	2	1
27	4	1	15	0	36	2	1
27	4	1	15	0	36	2	1
28	3	1	63	1	46	1	1
28	3	1	63	1	46	1	1
28	3	1	63	1	46	1	1
29	7	1	63	0	55	2	1
29	7	1	63	0	55	2	1
29	7	1	63	0	55	2	1
29	7	1	63	0	55	2	1
29	7	1	63	0	55	2	1
29	7	1	63	0	55	2	1
29	7	1	63	0	55	2	1
32	20	1	63	1	53	3	1
32	20	1	63	1	53	3	1
32	20	1	63	1	53	3	1
32	20	1	63	1	53	3	1
32	20	1	63	1	53	3	1
32	20	1	63	1	53	3	1
32	20	1	63	1	53	3	1
32	20	1	63	1	53	3	1
32	20	1	63	1	53	3	1
32	20	1	63	1	53	3	1
32	20	1	63	1	53	3	1
32	20	1	63	1	53	3	1
32	20	1	63	1	53	3	1
32	20	1	63	1	53	3	1
32	20	1	63	1	53	3	1
32	20	1	63	1	53	3	1
32	20	1	63	1	53	3	1
32	20	1	63	1	53	3	1
32	20	1	63	1	53	3	1
32	20	1	63	1	53	3	1
2	1	1	63	1	57	3	1
6	1	1	63	0	63	1	1
17	5	1	15	0	45	1	1
17	5	1	15	0	45	1	1
17	5	1	15	0	45	1	1
17	5	1	15	0	45	1	1
17	5	1	15	0	45	1	1
18	4	1	63	0	31	2	1
18	4	1	63	0	31	2	1
18	4	1	63	0	31	2	1
18	4	1	63	0	31	2	1
21	10	1	43	1	48	2	1
21	10	1	43	1	48	2	1
21	10	1	43	1	48	2	1
21	10	1	43	1	48	2	1

21	10	1	43	1	48	2	1
21	10	1	43	1	48	2	1
21	10	1	43	1	48	2	1
21	10	1	43	1	48	2	1
21	10	1	43	1	48	2	1
21	10	1	43	1	48	2	1
25	1	1	3	0	59	1	1
33	5	1	3	1	51	1	1
33	5	1	3	1	51	1	1
33	5	1	3	1	51	1	1
33	5	1	3	1	51	1	1
33	5	1	3	1	51	1	1
34	20	1	63	1	55	3	1
34	20	1	63	1	55	3	1
34	20	1	63	1	55	3	1
34	20	1	63	1	55	3	1
34	20	1	63	1	55	3	1
34	20	1	63	1	55	3	1
34	20	1	63	1	55	3	1
34	20	1	63	1	55	3	1
34	20	1	63	1	55	3	1
34	20	1	63	1	55	3	1
34	20	1	63	1	55	3	1
34	20	1	63	1	55	3	1
34	20	1	63	1	55	3	1
34	20	1	63	1	55	3	1
34	20	1	63	1	55	3	1
34	20	1	63	1	55	3	1
34	20	1	63	1	55	3	1
34	20	1	63	1	55	3	1
34	20	1	63	1	55	3	1
34	20	1	63	1	55	3	1
35	15	1	43	1	51	1	1
35	15	1	43	1	51	1	1
35	15	1	43	1	51	1	1
35	15	1	43	1	51	1	1
35	15	1	43	1	51	1	1
35	15	1	43	1	51	1	1
35	15	1	43	1	51	1	1
35	15	1	43	1	51	1	1
35	15	1	43	1	51	1	1
35	15	1	43	1	51	1	1
35	15	1	43	1	51	1	1
35	15	1	43	1	51	1	1
35	15	1	43	1	51	1	1
35	15	1	43	1	51	1	1
35	15	1	43	1	51	1	1
39	3	1	43	1	56	1	1
39	3	1	43	1	56	1	1
39	3	1	43	1	56	1	1

42	2	1	63	1	49	1	1
42	2	1	63	1	49	1	1
43	7	1	43	1	34	2	1
43	7	1	43	1	34	2	1
43	7	1	43	1	34	2	1
43	7	1	43	1	34	2	1
43	7	1	43	1	34	2	1
43	7	1	43	1	34	2	1
43	7	1	43	1	34	2	1
49	5	1	43	1	45	2	1
49	5	1	43	1	45	2	1
49	5	1	43	1	45	2	1
49	5	1	43	1	45	2	1
49	5	1	43	1	45	2	1
51	1	1	43	0	30	1	1
52	3	1	63	0	33	2	1
52	3	1	63	0	33	2	1
52	3	1	63	0	33	2	1
63	5	1	3	0	59	1	1
63	5	1	3	0	59	1	1
63	5	1	3	0	59	1	1
63	5	1	3	0	59	1	1
63	5	1	3	0	59	1	1
65	5	1	3	0	55	1	1
65	5	1	3	0	55	1	1
65	5	1	3	0	55	1	1
65	5	1	3	0	55	1	1
65	5	1	3	0	55	1	1
68	20	1	63	1	50	1	1
68	20	1	63	1	50	1	1
68	20	1	63	1	50	1	1
68	20	1	63	1	50	1	1
68	20	1	63	1	50	1	1
68	20	1	63	1	50	1	1
68	20	1	63	1	50	1	1
68	20	1	63	1	50	1	1
68	20	1	63	1	50	1	1
68	20	1	63	1	50	1	1
68	20	1	63	1	50	1	1
68	20	1	63	1	50	1	1
68	20	1	63	1	50	1	1
68	20	1	63	1	50	1	1
68	20	1	63	1	50	1	1
68	20	1	63	1	50	1	1
68	20	1	63	1	50	1	1
68	20	1	63	1	50	1	1
68	20	1	63	1	50	1	1
68	20	1	63	1	50	1	1
74	15	1	63	0	44	3	1
74	15	1	63	0	44	3	1

74	15	1	63	0	44	3	1
74	15	1	63	0	44	3	1
74	15	1	63	0	44	3	1
74	15	1	63	0	44	3	1
74	15	1	63	0	44	3	1
74	15	1	63	0	44	3	1
74	15	1	63	0	44	3	1
74	15	1	63	0	44	3	1
74	15	1	63	0	44	3	1
74	15	1	63	0	44	3	1
74	15	1	63	0	44	3	1
74	15	1	63	0	44	3	1
74	15	1	63	0	44	3	1
75	2	1	63	1	47	3	1
75	2	1	63	1	47	3	1
76	11	1	63	1	41	2	1
76	11	1	63	1	41	2	1
76	11	1	63	1	41	2	1
76	11	1	63	1	41	2	1
76	11	1	63	1	41	2	1
76	11	1	63	1	41	2	1
76	11	1	63	1	41	2	1
76	11	1	63	1	41	2	1
76	11	1	63	1	41	2	1
76	11	1	63	1	41	2	1
76	11	1	63	1	41	2	1
82	6	1	63	0	38	3	3
82	6	1	63	0	38	3	3
82	6	1	63	0	38	3	3
82	6	1	63	0	38	3	3
82	6	1	63	0	38	3	3
82	6	1	63	0	38	3	3
85	6	1	63	1	56	3	1
85	6	1	63	1	56	3	1
85	6	1	63	1	56	3	1
85	6	1	63	1	56	3	1
85	6	1	63	1	56	3	1
85	6	1	63	1	56	3	1
94	5	1	63	1	60	2	1
94	5	1	63	1	60	2	1
94	5	1	63	1	60	2	1
94	5	1	63	1	60	2	1
94	5	1	63	1	60	2	1
7	3	1	63	1	35	3	1
7	3	1	63	1	35	3	1
7	3	1	63	1	35	3	1
9	7	1	63	0	47	3	1
9	7	1	63	0	47	3	1
9	7	1	63	0	47	3	1
9	7	1	63	0	47	3	1

9	7	1	63	0	47	3	1
9	7	1	63	0	47	3	1
9	7	1	63	0	47	3	1
10	5	1	63	0	50	3	3
10	5	1	63	0	50	3	3
10	5	1	63	0	50	3	3
10	5	1	63	0	50	3	3
10	5	1	63	0	50	3	3
11	2	1	63	1	56	3	1
11	2	1	63	1	56	3	1
14	20	1	63	1	67	1	1
14	20	1	63	1	67	1	1
14	20	1	63	1	67	1	1
14	20	1	63	1	67	1	1
14	20	1	63	1	67	1	1
14	20	1	63	1	67	1	1
14	20	1	63	1	67	1	1
14	20	1	63	1	67	1	1
14	20	1	63	1	67	1	1
14	20	1	63	1	67	1	1
14	20	1	63	1	67	1	1
14	20	1	63	1	67	1	1
14	20	1	63	1	67	1	1
14	20	1	63	1	67	1	1
14	20	1	63	1	67	1	1
14	20	1	63	1	67	1	1
14	20	1	63	1	67	1	1
14	20	1	63	1	67	1	1
14	20	1	63	1	67	1	1
37	6	1	63	0	44	1	1
37	6	1	63	0	44	1	1
37	6	1	63	0	44	1	1
37	6	1	63	0	44	1	1
37	6	1	63	0	44	1	1
37	6	1	63	0	44	1	1
47	9	1	43	1	49	2	1
47	9	1	43	1	49	2	1
47	9	1	43	1	49	2	1
47	9	1	43	1	49	2	1
47	9	1	43	1	49	2	1
47	9	1	43	1	49	2	1
47	9	1	43	1	49	2	1
47	9	1	43	1	49	2	1
47	9	1	43	1	49	2	1
48	15	1	43	1	53	2	1
48	15	1	43	1	53	2	1
48	15	1	43	1	53	2	1
48	15	1	43	1	53	2	1
48	15	1	43	1	53	2	1

48	15	1	43	1	53	2	1
48	15	1	43	1	53	2	1
48	15	1	43	1	53	2	1
48	15	1	43	1	53	2	1
48	15	1	43	1	53	2	1
48	15	1	43	1	53	2	1
48	15	1	43	1	53	2	1
48	15	1	43	1	53	2	1
48	15	1	43	1	53	2	1
48	15	1	43	1	53	2	1
59	5	1	63	1	46	3	1
59	5	1	63	1	46	3	1
59	5	1	63	1	46	3	1
59	5	1	63	1	46	3	1
59	5	1	63	1	46	3	1
71	4	1	63	1	51	3	1
71	4	1	63	1	51	3	1
71	4	1	63	1	51	3	1
71	4	1	63	1	51	3	1
72	12	1	63	1	53	3	1
72	12	1	63	1	53	3	1
72	12	1	63	1	53	3	1
72	12	1	63	1	53	3	1
72	12	1	63	1	53	3	1
72	12	1	63	1	53	3	1
72	12	1	63	1	53	3	1
72	12	1	63	1	53	3	1
72	12	1	63	1	53	3	1
72	12	1	63	1	53	3	1
72	12	1	63	1	53	3	1
72	12	1	63	1	53	3	1
80	20	1	63	1	58	3	1
80	20	1	63	1	58	3	1
80	20	1	63	1	58	3	1
80	20	1	63	1	58	3	1
80	20	1	63	1	58	3	1
80	20	1	63	1	58	3	1
80	20	1	63	1	58	3	1
80	20	1	63	1	58	3	1
80	20	1	63	1	58	3	1
80	20	1	63	1	58	3	1
80	20	1	63	1	58	3	1
80	20	1	63	1	58	3	1
80	20	1	63	1	58	3	1
80	20	1	63	1	58	3	1
80	20	1	63	1	58	3	1
80	20	1	63	1	58	3	1
80	20	1	63	1	58	3	1
80	20	1	63	1	58	3	1
80	20	1	63	1	58	3	1

80	20	1	63	1	58	3	1
81	8	1	63	1	46	3	2
81	8	1	63	1	46	3	2
81	8	1	63	1	46	3	2
81	8	1	63	1	46	3	2
81	8	1	63	1	46	3	2
81	8	1	63	1	46	3	2
81	8	1	63	1	46	3	2
81	8	1	63	1	46	3	2
86	5	1	63	0	49	3	1
86	5	1	63	0	49	3	1
86	5	1	63	0	49	3	1
86	5	1	63	0	49	3	1
86	5	1	63	0	49	3	1
87	5	1	63	0	47	3	1
87	5	1	63	0	47	3	1
87	5	1	63	0	47	3	1
87	5	1	63	0	47	3	1
87	5	1	63	0	47	3	1
89	11	1	63	1	50	3	1
89	11	1	63	1	50	3	1
89	11	1	63	1	50	3	1
89	11	1	63	1	50	3	1
89	11	1	63	1	50	3	1
89	11	1	63	1	50	3	1
89	11	1	63	1	50	3	1
89	11	1	63	1	50	3	1
89	11	1	63	1	50	3	1
89	11	1	63	1	50	3	1
89	11	1	63	1	50	3	1
90	11	1	63	0	48	2	1
90	11	1	63	0	48	2	1
90	11	1	63	0	48	2	1
90	11	1	63	0	48	2	1
90	11	1	63	0	48	2	1
90	11	1	63	0	48	2	1
90	11	1	63	0	48	2	1
90	11	1	63	0	48	2	1
90	11	1	63	0	48	2	1
90	11	1	63	0	48	2	1
90	11	1	63	0	48	2	1
98	2	1	15	1	48	1	1
98	2	1	15	1	48	1	1
100	19	1	63	0	52	3	3
100	19	1	63	0	52	3	3
100	19	1	63	0	52	3	3
100	19	1	63	0	52	3	3
100	19	1	63	0	52	3	3
100	19	1	63	0	52	3	3
100	19	1	63	0	52	3	3

100	19	1	63	0	52	3	3
100	19	1	63	0	52	3	3
100	19	1	63	0	52	3	3
100	19	1	63	0	52	3	3
100	19	1	63	0	52	3	3
100	19	1	63	0	52	3	3
100	19	1	63	0	52	3	3
100	19	1	63	0	52	3	3
100	19	1	63	0	52	3	3
100	19	1	63	0	52	3	3
100	19	1	63	0	52	3	3
100	19	1	63	0	52	3	3
102	8	1	63	1	50	3	1
102	8	1	63	1	50	3	1
102	8	1	63	1	50	3	1
102	8	1	63	1	50	3	1
102	8	1	63	1	50	3	1
102	8	1	63	1	50	3	1
102	8	1	63	1	50	3	1
102	8	1	63	1	50	3	1

sport_med	quest_sport	pres_marche	pres_stoptbc	pres_regime	pres_sport	temps_educ
0	1	6	6	6	6	4
0	1	6	6	6	6	4
0	1	6	6	6	6	4
0	1	6	6	6	6	4
0	1	7	7	7	7	7
0	1	7	7	7	7	7
0	1	7	7	7	7	7
0	1	5	7	7	7	7
0	1	5	7	7	7	7
0	1	5	7	7	7	7
0	1	5	7	7	7	7
0	1	5	7	7	7	7
0	1	5	7	7	7	7
0	1	5	7	7	7	7
0	1	4	7	7	4	3
0	1	4	7	7	4	3
0	1	4	7	7	4	3
0	1	4	7	7	4	3
0	1	4	7	7	4	3
0	1	4	7	7	4	3
0	1	4	7	7	4	3
0	1	4	7	7	4	3
0	1	4	7	7	4	3
0	1	4	7	7	4	3
0	1	4	7	7	4	3
0	1	4	7	7	4	3
0	1	4	7	7	4	3
0	1	4	7	7	4	3
0	1	4	7	7	4	3
0	1	4	7	7	4	3
0	1	4	7	7	4	3
0	1	4	7	7	4	3
0	1	4	7	7	4	3
0	1	4	7	7	4	3
1	1	7	5	7	7	7
1	1	5	5	7	5	6
1	1	7	6	6	7	5
1	1	7	6	6	7	5
1	1	7	6	6	7	5
1	1	7	6	6	7	5
1	1	7	6	6	7	5
1	1	7	3	5	7	4
1	1	7	3	5	7	4
1	1	7	3	5	7	4
1	1	7	3	5	7	4
1	1	5	5	6	5	5
1	1	5	5	6	5	5
1	1	5	5	6	5	5
1	1	5	5	6	5	5

1	1	5	5	6	5	5
1	1	5	5	6	5	5
1	1	5	5	6	5	5
1	1	5	5	6	5	5
1	1	5	5	6	5	5
1	1	5	5	6	5	5
1	1	7	7	7	7	6
1	1	7	7	7	7	5
1	1	7	7	7	7	5
1	1	7	7	7	7	5
1	1	7	7	7	7	5
1	1	7	7	7	7	5
1	1	7	4	7	7	2
1	1	7	4	7	7	2
1	1	7	4	7	7	2
1	1	7	4	7	7	2
1	1	7	4	7	7	2
1	1	7	4	7	7	2
1	1	7	4	7	7	2
1	1	7	4	7	7	2
1	1	7	4	7	7	2
1	1	7	4	7	7	2
1	1	7	4	7	7	2
1	1	7	4	7	7	2
1	1	7	4	7	7	2
1	1	7	4	7	7	2
1	1	7	4	7	7	2
1	1	7	4	7	7	2
1	1	7	4	7	7	2
1	1	7	4	7	7	2
1	1	7	4	7	7	2
1	1	7	4	7	7	2
1	1	6	7	7	7	6
1	1	6	7	7	7	6
1	1	6	7	7	7	6
1	1	6	7	7	7	6
1	1	6	7	7	7	6
1	1	6	7	7	7	6
1	1	6	7	7	7	6
1	1	6	7	7	7	6
1	1	6	7	7	7	6
1	1	6	7	7	7	6
1	1	6	7	7	7	6
1	1	6	7	7	7	6
1	1	6	7	7	7	6
1	1	6	7	7	7	6
1	1	6	7	7	7	6
1	1	6	5	6	6	4
1	1	6	5	6	6	4
1	1	6	5	6	6	4

1	1	7	7	7	7	7
1	1	7	7	7	7	7
1	1	4	7	5	4	4
1	1	4	7	5	4	4
1	1	4	7	5	4	4
1	1	4	7	5	4	4
1	1	4	7	5	4	4
1	1	4	7	5	4	4
1	1	4	7	5	4	4
1	1	5	6	4	5	3
1	1	5	6	4	5	3
1	1	5	6	4	5	3
1	1	5	6	4	5	3
1	1	5	6	4	5	3
1	1	4	5	7	5	4
1	1	7	7	7	7	6
1	1	7	7	7	7	6
1	1	7	7	7	7	6
1	1	5	6	7	7	6
1	1	5	6	7	7	6
1	1	5	6	7	7	6
1	1	5	6	7	7	6
1	1	5	6	7	7	6
1	1	6	4	6	6	5
1	1	6	4	6	6	5
1	1	6	4	6	6	5
1	1	6	4	6	6	5
1	1	6	4	6	6	5
1	1	4	6	6	5	3
1	1	4	6	6	5	3
1	1	4	6	6	5	3
1	1	4	6	6	5	3
1	1	4	6	6	5	3
1	1	4	6	6	5	3
1	1	4	6	6	5	3
1	1	4	6	6	5	3
1	1	4	6	6	5	3
1	1	4	6	6	5	3
1	1	4	6	6	5	3
1	1	4	6	6	5	3
1	1	4	6	6	5	3
1	1	4	6	6	5	3
1	1	4	6	6	5	3
1	1	4	6	6	5	3
1	1	4	6	6	5	3
1	1	4	6	6	5	3
1	1	4	6	6	5	3
1	1	4	6	6	5	3
1	1	7	3	7	7	1
1	1	7	3	7	7	1

1	1	7	3	7	7	1
1	1	7	3	7	7	1
1	1	7	3	7	7	1
1	1	7	3	7	7	1
1	1	7	3	7	7	1
1	1	7	3	7	7	1
1	1	7	3	7	7	1
1	1	7	3	7	7	1
1	1	7	3	7	7	1
1	1	7	3	7	7	1
1	1	7	3	7	7	1
1	1	7	3	7	7	1
1	1	7	3	7	7	1
1	1	7	7	7	7	6
1	1	7	7	7	7	6
1	1	6	4	7	6	5
1	1	6	4	7	6	5
1	1	6	4	7	6	5
1	1	6	4	7	6	5
1	1	6	4	7	6	5
1	1	6	4	7	6	5
1	1	6	4	7	6	5
1	1	6	4	7	6	5
1	1	6	4	7	6	5
1	1	6	4	7	6	5
1	1	6	4	7	6	5
1	1	7	3	7	5	6
1	1	7	3	7	5	6
1	1	7	3	7	5	6
1	1	7	3	7	5	6
1	1	7	3	7	5	6
1	1	7	3	7	5	6
1	1	7	7	7	7	7
1	1	7	7	7	7	7
1	1	7	7	7	7	7
1	1	7	7	7	7	7
1	1	7	7	7	7	7
1	1	7	7	7	7	7
1	1	7	7	7	7	7
1	1	7	7	7	7	7
1	1	7	7	7	7	7
1	1	7	7	7	7	7
1	1	7	7	7	7	7
2	1	7	5	7	7	7
2	1	7	5	7	7	7
2	1	7	5	7	7	7
2	1	6	6	6	6	6
2	1	6	6	6	6	6
2	1	6	6	6	6	6
2	1	6	6	6	6	6

2	1	6	6	6	6	6
2	1	6	6	6	6	6
2	1	6	6	6	6	6
2	1	7	3	6	7	5
2	1	7	3	6	7	5
2	1	7	3	6	7	5
2	1	7	3	6	7	5
2	1	7	3	6	7	5
2	1	7	7	7	7	5
2	1	7	7	7	7	5
2	0	3	1	5	2	2
2	0	3	1	5	2	2
2	0	3	1	5	2	2
2	0	3	1	5	2	2
2	0	3	1	5	2	2
2	0	3	1	5	2	2
2	0	3	1	5	2	2
2	0	3	1	5	2	2
2	0	3	1	5	2	2
2	0	3	1	5	2	2
2	0	3	1	5	2	2
2	0	3	1	5	2	2
2	0	3	1	5	2	2
2	0	3	1	5	2	2
2	0	3	1	5	2	2
2	0	3	1	5	2	2
2	0	3	1	5	2	2
2	0	3	1	5	2	2
2	0	3	1	5	2	2
2	0	3	1	5	2	2
2	1	7	1	7	7	6
2	1	7	1	7	7	6
2	1	7	1	7	7	6
2	1	7	1	7	7	6
2	1	7	1	7	7	6
2	1	7	1	7	7	6
2	1	6	7	7	7	7
2	1	6	7	7	7	7
2	1	6	7	7	7	7
2	1	6	7	7	7	7
2	1	6	7	7	7	7
2	1	6	7	7	7	7
2	1	6	7	7	7	7
2	1	6	7	7	7	7
2	1	6	7	7	7	7
2	1	6	7	7	6	5
2	1	6	7	7	6	5
2	1	6	7	7	6	5
2	1	6	7	7	6	5
2	1	6	7	7	6	5

2	1	6	7	7	6	5
2	1	6	7	7	6	5
2	1	6	7	7	6	5
2	1	6	7	7	6	5
2	1	6	7	7	6	5
2	1	6	7	7	6	5
2	1	6	7	7	6	5
2	1	6	7	7	6	5
2	1	6	7	7	6	5
2	1	6	7	7	6	5
2	1	6	7	7	7	4
2	1	6	7	7	7	4
2	1	6	7	7	7	4
2	1	6	7	7	7	4
2	1	6	7	7	7	4
2	1	7	6	6	6	6
2	1	7	6	6	6	6
2	1	7	6	6	6	6
2	1	7	6	6	6	6
2	1	7	6	7	7	7
2	1	7	6	7	7	7
2	1	7	6	7	7	7
2	1	7	6	7	7	7
2	1	7	6	7	7	7
2	1	7	6	7	7	7
2	1	7	6	7	7	7
2	1	7	6	7	7	7
2	1	7	6	7	7	7
2	1	7	6	7	7	7
2	1	7	6	7	7	7
2	1	7	6	7	7	7
2	1	7	7	6	7	5
2	1	7	7	6	7	5
2	1	7	7	6	7	5
2	1	7	7	6	7	5
2	1	7	7	6	7	5
2	1	7	7	6	7	5
2	1	7	7	6	7	5
2	1	7	7	6	7	5
2	1	7	7	6	7	5
2	1	7	7	6	7	5
2	1	7	7	6	7	5
2	1	7	7	6	7	5
2	1	7	7	6	7	5
2	1	7	7	6	7	5
2	1	7	7	6	7	5
2	1	7	7	6	7	5
2	1	7	7	6	7	5
2	1	7	7	6	7	5
2	1	7	7	6	7	5

2	1	7	7	6	7	5
2	1	7	7	7	7	7
2	1	7	7	7	7	7
2	1	7	7	7	7	7
2	1	7	7	7	7	7
2	1	7	7	7	7	7
2	1	7	7	7	7	7
2	1	7	7	7	7	7
2	1	7	7	7	7	7
2	1	7	7	7	7	7
2	1	7	7	7	7	7
2	1	7	7	7	7	7
2	1	7	7	7	7	7
2	1	7	7	7	7	7
2	1	5	7	6	5	6
2	1	5	7	6	5	6
2	1	5	7	6	5	6
2	1	5	7	6	5	6
2	1	5	7	6	5	6
2	1	7	7	7	7	7
2	1	7	7	7	7	7
2	1	7	7	7	7	7
2	1	7	7	7	7	7
2	1	7	7	7	7	7
2	1	7	7	7	7	7
2	1	7	7	7	7	7
2	1	7	7	7	7	7
2	1	7	7	7	7	7
2	1	7	7	7	7	7
2	1	7	7	7	7	7
2	1	5	6	7	5	5
2	1	5	6	7	5	5
2	1	5	6	7	5	5
2	1	5	6	7	5	5
2	1	5	6	7	5	5
2	1	5	6	7	5	5
2	1	5	6	7	5	5
2	1	5	6	7	5	5
2	1	5	6	7	5	5
2	1	5	6	7	5	5
2	1	5	6	7	5	5
2	1	6	7	5	6	6
2	1	6	7	5	6	6
2	1	7	6	7	7	6
2	1	7	6	7	7	6
2	1	7	6	7	7	6
2	1	7	6	7	7	6
2	1	7	6	7	7	6
2	1	7	6	7	7	6
2	1	7	6	7	7	6

2	1	7	6	7	7	6
2	1	7	6	7	7	6
2	1	7	6	7	7	6
2	1	7	6	7	7	6
2	1	7	6	7	7	6
2	1	7	6	7	7	6
2	1	7	6	7	7	6
2	1	7	6	7	7	6
2	1	7	6	7	7	6
2	1	7	6	7	7	6
2	1	7	6	7	7	6
2	1	7	6	7	7	6
2	1	6	6	6	6	5
2	1	6	6	6	6	5
2	1	6	6	6	6	5
2	1	6	6	6	6	5
2	1	6	6	6	6	5
2	1	6	6	6	6	5
2	1	6	6	6	6	5
2	1	6	6	6	6	5

priomed_rhd	priomed_ado1	priomed_ado2	temps_sport	fmc_diabete	fmc_sport	fmc_nutri
5	5	0	1	1	1	1
5	5	0	1	1	1	1
5	5	0	1	1	1	1
5	5	0	1	1	1	1
4	5	1	1	1	1	1
4	5	1	1	1	1	1
4	5	1	1	1	1	1
10	0	0	1	1	0	1
10	0	0	1	1	0	1
10	0	0	1	1	0	1
10	0	0	1	1	0	1
10	0	0	1	1	0	1
10	0	0	1	1	0	1
10	0	0	1	1	0	1
4	6	0	1	1	0	1
4	6	0	1	1	0	1
4	6	0	1	1	0	1
4	6	0	1	1	0	1
4	6	0	1	1	0	1
4	6	0	1	1	0	1
4	6	0	1	1	0	1
4	6	0	1	1	0	1
4	6	0	1	1	0	1
4	6	0	1	1	0	1
4	6	0	1	1	0	1
4	6	0	1	1	0	1
4	6	0	1	1	0	1
4	6	0	1	1	0	1
4	6	0	1	1	0	1
4	6	0	1	1	0	1
4	6	0	1	1	0	1
4	6	0	1	1	0	1
4	6	0	1	1	0	1
4	6	0	1	1	0	1
10	0	0	1	1	1	1
8	2	0	1	1	0	1
10	0	0	1	1	1	1
10	0	0	1	1	1	1
10	0	0	1	1	1	1
10	0	0	1	1	1	1
10	0	0	1	1	1	1
7	3	0	1	1	0	1
7	3	0	1	1	0	1
7	3	0	1	1	0	1
7	3	0	1	1	0	1
5	5	0	1	1	1	1
5	5	0	1	1	1	1
5	5	0	1	1	1	1
5	5	0	1	1	1	1

5	5	0	1	1	1	1
5	5	0	1	1	1	1
5	5	0	1	1	1	1
5	5	0	1	1	1	1
5	5	0	1	1	1	1
5	5	0	1	1	1	1
5	5	0	1	1	1	1
1	4	5	1	1	1	1
1	4	5	1	1	1	1
1	4	5	1	1	1	1
1	4	5	1	1	1	1
1	4	5	1	1	1	1
5	3	2	1	1	1	1
5	3	2	1	1	1	1
5	3	2	1	1	1	1
5	3	2	1	1	1	1
5	3	2	1	1	1	1
5	3	2	1	1	1	1
5	3	2	1	1	1	1
5	3	2	1	1	1	1
5	3	2	1	1	1	1
5	3	2	1	1	1	1
5	3	2	1	1	1	1
5	3	2	1	1	1	1
5	3	2	1	1	1	1
5	3	2	1	1	1	1
5	3	2	1	1	1	1
5	3	2	1	1	1	1
5	3	2	1	1	1	1
5	3	2	1	1	1	1
5	3	2	1	1	1	1
10	0	0	2	1	1	1
10	0	0	2	1	1	1
10	0	0	2	1	1	1
10	0	0	2	1	1	1
10	0	0	2	1	1	1
10	0	0	2	1	1	1
10	0	0	2	1	1	1
10	0	0	2	1	1	1
10	0	0	2	1	1	1
10	0	0	2	1	1	1
10	0	0	2	1	1	1
10	0	0	2	1	1	1
10	0	0	2	1	1	1
10	0	0	2	1	1	1
10	0	0	2	1	1	1
4	3	3	1	1	1	0
4	3	3	1	1	1	0
4	3	3	1	1	1	0

4	4	2	2	1	1	1
4	4	2	2	1	1	1
5	5	0	1	1	0	0
5	5	0	1	1	0	0
5	5	0	1	1	0	0
5	5	0	1	1	0	0
5	5	0	1	1	0	0
5	5	0	1	1	0	0
5	5	0	1	1	0	0
6	4	0	1	0	0	0
6	4	0	1	0	0	0
6	4	0	1	0	0	0
6	4	0	1	0	0	0
6	4	0	1	0	0	0
6	4	0	1	1	1	1
8	2	0	1	0	0	0
8	2	0	1	0	0	0
8	2	0	1	0	0	0
8	2	0	1	1	0	1
8	2	0	1	1	0	1
8	2	0	1	1	0	1
8	2	0	1	1	0	1
8	2	0	1	1	0	1
8	2	0	1	1	0	1
8	2	0	1	1	0	1
8	2	0	1	1	0	1
8	2	0	1	1	0	1
8	2	0	1	1	0	1
5	3	2	1	1	0	1
5	3	2	1	1	0	1
5	3	2	1	1	0	1
5	3	2	1	1	0	1
5	3	2	1	1	0	1
5	3	2	1	1	0	1
5	3	2	1	1	0	1
5	3	2	1	1	0	1
5	3	2	1	1	0	1
5	3	2	1	1	0	1
5	3	2	1	1	0	1
5	3	2	1	1	0	1
5	3	2	1	1	0	1
5	3	2	1	1	0	1
5	3	2	1	1	0	1
5	3	2	1	1	0	1
5	3	2	1	1	0	1
5	3	2	1	1	0	1
5	3	2	1	1	0	1
5	3	2	1	1	0	1
7	3	0	1	0	0	0
7	3	0	1	0	0	0

7	3	0	1	0	0	0
7	3	0	1	0	0	0
7	3	0	1	0	0	0
7	3	0	1	0	0	0
7	3	0	1	0	0	0
7	3	0	1	0	0	0
7	3	0	1	0	0	0
7	3	0	1	0	0	0
7	3	0	1	0	0	0
7	3	0	1	0	0	0
7	3	0	1	0	0	0
7	3	0	1	0	0	0
7	3	0	1	0	0	0
9	1	0	2	1	1	1
9	1	0	2	1	1	1
7	3	0	1	1	1	1
7	3	0	1	1	1	1
7	3	0	1	1	1	1
7	3	0	1	1	1	1
7	3	0	1	1	1	1
7	3	0	1	1	1	1
7	3	0	1	1	1	1
7	3	0	1	1	1	1
7	3	0	1	1	1	1
7	3	0	1	1	1	1
7	3	0	1	1	1	1
9	1	0	1	1	0	1
9	1	0	1	1	0	1
9	1	0	1	1	0	1
9	1	0	1	1	0	1
9	1	0	1	1	0	1
9	1	0	1	1	0	1
7	2	1	2	1	1	1
7	2	1	2	1	1	1
7	2	1	2	1	1	1
7	2	1	2	1	1	1
7	2	1	2	1	1	1
7	2	1	2	1	1	1
5	5	0	1	1	1	1
5	5	0	1	1	1	1
5	5	0	1	1	1	1
5	5	0	1	1	1	1
5	5	0	1	1	1	1
10	0	0	1	1	0	0
10	0	0	1	1	0	0
10	0	0	1	1	0	0
4	4	2	1	1	1	1
4	4	2	1	1	1	1
4	4	2	1	1	1	1
4	4	2	1	1	1	1

4	4	2	1	1	1	1
4	4	2	1	1	1	1
4	4	2	1	1	1	1
10	0	0	1	1	0	0
10	0	0	1	1	0	0
10	0	0	1	1	0	0
10	0	0	1	1	0	0
10	0	0	1	1	0	0
4	4	2	1	0	0	0
4	4	2	1	0	0	0
7	3	0	1	0	0	0
7	3	0	1	0	0	0
7	3	0	1	0	0	0
7	3	0	1	0	0	0
7	3	0	1	0	0	0
7	3	0	1	0	0	0
7	3	0	1	0	0	0
7	3	0	1	0	0	0
7	3	0	1	0	0	0
7	3	0	1	0	0	0
7	3	0	1	0	0	0
7	3	0	1	0	0	0
7	3	0	1	0	0	0
7	3	0	1	0	0	0
7	3	0	1	0	0	0
7	3	0	1	0	0	0
7	3	0	1	0	0	0
7	3	0	1	0	0	0
7	3	0	1	0	0	0
7	3	0	1	0	0	0
10	0	0	2	1	1	1
10	0	0	2	1	1	1
10	0	0	2	1	1	1
10	0	0	2	1	1	1
10	0	0	2	1	1	1
10	0	0	2	1	1	1
5	3	2	1	1	1	1
5	3	2	1	1	1	1
5	3	2	1	1	1	1
5	3	2	1	1	1	1
5	3	2	1	1	1	1
5	3	2	1	1	1	1
5	3	2	1	1	1	1
5	3	2	1	1	1	1
5	3	2	1	1	1	1
4	3	3	1	1	0	1
4	3	3	1	1	0	1
4	3	3	1	1	0	1
4	3	3	1	1	0	1
4	3	3	1	1	0	1

4	3	3	1	1	0	1
4	3	3	1	1	0	1
4	3	3	1	1	0	1
4	3	3	1	1	0	1
4	3	3	1	1	0	1
4	3	3	1	1	0	1
4	3	3	1	1	0	1
4	3	3	1	1	0	1
4	3	3	1	1	0	1
4	3	3	1	1	0	1
6	4	0	1	1	0	1
6	4	0	1	1	0	1
6	4	0	1	1	0	1
6	4	0	1	1	0	1
6	4	0	1	1	0	1
6	4	0	1	0	0	0
6	4	0	1	0	0	0
6	4	0	1	0	0	0
6	4	0	1	0	0	0
5	3	2	1	1	1	1
5	3	2	1	1	1	1
5	3	2	1	1	1	1
5	3	2	1	1	1	1
5	3	2	1	1	1	1
5	3	2	1	1	1	1
5	3	2	1	1	1	1
5	3	2	1	1	1	1
5	3	2	1	1	1	1
5	3	2	1	1	1	1
5	3	2	1	1	1	1
5	3	2	1	1	1	1
4	3	3	1	1	0	0
4	3	3	1	1	0	0
4	3	3	1	1	0	0
4	3	3	1	1	0	0
4	3	3	1	1	0	0
4	3	3	1	1	0	0
4	3	3	1	1	0	0
4	3	3	1	1	0	0
4	3	3	1	1	0	0
4	3	3	1	1	0	0
4	3	3	1	1	0	0
4	3	3	1	1	0	0
4	3	3	1	1	0	0
4	3	3	1	1	0	0
4	3	3	1	1	0	0
4	3	3	1	1	0	0
4	3	3	1	1	0	0
4	3	3	1	1	0	0
4	3	3	1	1	0	0

4	3	3	1	1	0	0
8	1	1	2	1	1	1
8	1	1	2	1	1	1
8	1	1	2	1	1	1
8	1	1	2	1	1	1
8	1	1	2	1	1	1
8	1	1	2	1	1	1
8	1	1	2	1	1	1
8	1	1	2	1	1	1
7	2	1	1	1	1	1
7	2	1	1	1	1	1
7	2	1	1	1	1	1
7	2	1	1	1	1	1
7	2	1	1	1	1	1
7	3	0	1	1	1	1
7	3	0	1	1	1	1
7	3	0	1	1	1	1
7	3	0	1	1	1	1
7	3	0	1	1	1	1
3	1	6	2	1	1	1
3	1	6	2	1	1	1
3	1	6	2	1	1	1
3	1	6	2	1	1	1
3	1	6	2	1	1	1
3	1	6	2	1	1	1
3	1	6	2	1	1	1
3	1	6	2	1	1	1
3	1	6	2	1	1	1
3	1	6	2	1	1	1
3	1	6	2	1	1	1
7	3	0	1	0	0	0
7	3	0	1	0	0	0
7	3	0	1	0	0	0
7	3	0	1	0	0	0
7	3	0	1	0	0	0
7	3	0	1	0	0	0
7	3	0	1	0	0	0
7	3	0	1	0	0	0
7	3	0	1	0	0	0
7	3	0	1	0	0	0
7	3	0	1	0	0	0
3	6	1	1	0	0	0
3	6	1	1	0	0	0
8	2	0	1	0	0	0
8	2	0	1	0	0	0
8	2	0	1	0	0	0
8	2	0	1	0	0	0
8	2	0	1	0	0	0
8	2	0	1	0	0	0
8	2	0	1	0	0	0

8	2	0	1	0	0	0
8	2	0	1	0	0	0
8	2	0	1	0	0	0
8	2	0	1	0	0	0
8	2	0	1	0	0	0
8	2	0	1	0	0	0
8	2	0	1	0	0	0
8	2	0	1	0	0	0
8	2	0	1	0	0	0
8	2	0	1	0	0	0
8	2	0	1	0	0	0
8	2	0	1	0	0	0
4	4	2	2	1	1	1
4	4	2	2	1	1	1
4	4	2	2	1	1	1
4	4	2	2	1	1	1
4	4	2	2	1	1	1
4	4	2	2	1	1	1
4	4	2	2	1	1	1
4	4	2	2	1	1	1

fmc_pharma	shtfmc_sport	bm_desequilibre	bm_hypoglycemie	bm_fatigue	bm_blessure
1	0	2	4	1	1
1	0	2	4	1	1
1	0	2	4	1	1
1	0	2	4	1	1
1	0	1	1	1	1
1	0	1	1	1	1
1	0	1	1	1	1
1	1	1	1	1	1
1	1	1	1	1	1
1	1	1	1	1	1
1	1	1	1	1	1
1	1	1	1	1	1
1	1	1	1	1	1
1	1	1	1	1	1
1	0	4	2	4	4
1	0	4	2	4	4
1	0	4	2	4	4
1	0	4	2	4	4
1	0	4	2	4	4
1	0	4	2	4	4
1	0	4	2	4	4
1	0	4	2	4	4
1	0	4	2	4	4
1	0	4	2	4	4
1	0	4	2	4	4
1	0	4	2	4	4
1	0	4	2	4	4
1	0	4	2	4	4
1	0	4	2	4	4
1	0	4	2	4	4
1	0	4	2	4	4
1	0	4	2	4	4
1	0	4	2	4	4
1	0	4	2	4	4
1	1	1	1	1	3
1	0	7	5	1	5
0	0	2	2	1	1
0	0	2	2	1	1
0	0	2	2	1	1
0	0	2	2	1	1
0	0	2	2	1	1
1	1	1	3	2	2
1	1	1	3	2	2
1	1	1	3	2	2
1	1	1	3	2	2
1	1	2	2	2	2
1	1	2	2	2	2
1	1	2	2	2	2
1	1	2	2	2	2

1	1	2	2	2	2
1	1	2	2	2	2
1	1	2	2	2	2
1	1	2	2	2	2
1	1	2	2	2	2
1	1	2	2	2	2
1	0	1	1	1	1
1	1	1	3	4	1
1	1	1	3	4	1
1	1	1	3	4	1
1	1	1	3	4	1
1	1	1	3	4	1
1	0	1	6	1	1
1	0	1	6	1	1
1	0	1	6	1	1
1	0	1	6	1	1
1	0	1	6	1	1
1	0	1	6	1	1
1	0	1	6	1	1
1	0	1	6	1	1
1	0	1	6	1	1
1	0	1	6	1	1
1	0	1	6	1	1
1	0	1	6	1	1
1	0	1	6	1	1
1	0	1	6	1	1
1	0	1	6	1	1
1	0	1	6	1	1
1	0	1	6	1	1
1	0	1	6	1	1
1	0	1	6	1	1
1	0	1	1	1	1
1	0	1	1	1	1
1	0	1	1	1	1
1	0	1	1	1	1
1	0	1	1	1	1
1	0	1	1	1	1
1	0	1	1	1	1
1	0	1	1	1	1
1	0	1	1	1	1
1	0	1	1	1	1
1	0	1	1	1	1
1	0	1	1	1	1
1	0	1	1	1	1
1	0	1	1	1	1
1	0	1	1	1	1
1	1	2	2	1	1
1	1	2	2	1	1
1	1	2	2	1	1

1	1	5	7	2	2
1	1	5	7	2	2
1	1	1	1	1	3
1	1	1	1	1	3
1	1	1	1	1	3
1	1	1	1	1	3
1	1	1	1	1	3
1	1	1	1	1	3
1	1	1	1	1	3
0	0	1	3	1	1
0	0	1	3	1	1
0	0	1	3	1	1
0	0	1	3	1	1
0	0	1	3	1	1
1	1	1	1	1	1
0	1	1	2	1	2
0	1	1	2	1	2
0	1	1	2	1	2
1	0	1	2	2	1
1	0	1	2	2	1
1	0	1	2	2	1
1	0	1	2	2	1
1	0	1	2	2	1
0	1	1	3	2	1
0	1	1	3	2	1
0	1	1	3	2	1
0	1	1	3	2	1
0	1	1	3	2	1
1	1	1	5	5	2
1	1	1	5	5	2
1	1	1	5	5	2
1	1	1	5	5	2
1	1	1	5	5	2
1	1	1	5	5	2
1	1	1	5	5	2
1	1	1	5	5	2
1	1	1	5	5	2
1	1	1	5	5	2
1	1	1	5	5	2
1	1	1	5	5	2
1	1	1	5	5	2
1	1	1	5	5	2
1	1	1	5	5	2
1	1	1	5	5	2
1	1	1	5	5	2
1	1	1	5	5	2
1	1	1	5	5	2
1	1	1	5	5	2
0	0	1	4	4	1
0	0	1	4	4	1

0	0	1	4	4	1
0	0	1	4	4	1
0	0	1	4	4	1
0	0	1	4	4	1
0	0	1	4	4	1
0	0	1	4	4	1
0	0	1	4	4	1
0	0	1	4	4	1
0	0	1	4	4	1
0	0	1	4	4	1
0	0	1	4	4	1
0	0	1	4	4	1
0	0	1	4	4	1
1	0	1	1	1	1
1	0	1	1	1	1
0	1	3	4	2	2
0	1	3	4	2	2
0	1	3	4	2	2
0	1	3	4	2	2
0	1	3	4	2	2
0	1	3	4	2	2
0	1	3	4	2	2
0	1	3	4	2	2
0	1	3	4	2	2
0	1	3	4	2	2
0	1	3	4	2	2
1	1	1	2	1	3
1	1	1	2	1	3
1	1	1	2	1	3
1	1	1	2	1	3
1	1	1	2	1	3
1	1	1	2	1	3
1	0	1	4	1	1
1	0	1	4	1	1
1	0	1	4	1	1
1	0	1	4	1	1
1	0	1	4	1	1
1	0	1	4	1	1
1	0	1	2	1	1
1	0	1	2	1	1
1	0	1	2	1	1
1	0	1	2	1	1
1	0	1	2	1	1
1	0	2	3	3	3
1	0	2	3	3	3
1	0	2	3	3	3
1	0	1	2	1	1
1	0	1	2	1	1
1	0	1	2	1	1
1	0	1	2	1	1

1	0	1	2	1	1
1	0	1	2	1	1
1	0	1	2	1	1
1	0	1	2	2	2
1	0	1	2	2	2
1	0	1	2	2	2
1	0	1	2	2	2
1	0	1	2	2	2
0	0	1	1	1	4
0	0	1	1	1	4
0	0	1	2	2	1
0	0	1	2	2	1
0	0	1	2	2	1
0	0	1	2	2	1
0	0	1	2	2	1
0	0	1	2	2	1
0	0	1	2	2	1
0	0	1	2	2	1
0	0	1	2	2	1
0	0	1	2	2	1
0	0	1	2	2	1
0	0	1	2	2	1
0	0	1	2	2	1
0	0	1	2	2	1
0	0	1	2	2	1
0	0	1	2	2	1
0	0	1	2	2	1
0	0	1	2	2	1
0	0	1	2	2	1
0	0	1	2	2	1
1	0	1	1	1	1
1	0	1	1	1	1
1	0	1	1	1	1
1	0	1	1	1	1
1	0	1	1	1	1
1	0	1	1	1	1
1	0	1	1	1	2
1	0	1	1	1	2
1	0	1	1	1	2
1	0	1	1	1	2
1	0	1	1	1	2
1	0	1	1	1	2
1	0	1	1	1	2
1	0	1	1	1	2
1	0	1	1	1	2
1	1	4	6	4	1
1	1	4	6	4	1
1	1	4	6	4	1
1	1	4	6	4	1
1	1	4	6	4	1

1	1	4	6	4	1
1	1	4	6	4	1
1	1	4	6	4	1
1	1	4	6	4	1
1	1	4	6	4	1
1	1	4	6	4	1
1	1	4	6	4	1
1	1	4	6	4	1
1	1	4	6	4	1
1	1	4	6	4	1
1	1	3	1	2	3
1	1	3	1	2	3
1	1	3	1	2	3
1	1	3	1	2	3
1	1	3	1	2	3
0	0	2	2	2	2
0	0	2	2	2	2
0	0	2	2	2	2
0	0	2	2	2	2
1	0	1	1	1	4
1	0	1	1	1	4
1	0	1	1	1	4
1	0	1	1	1	4
1	0	1	1	1	4
1	0	1	1	1	4
1	0	1	1	1	4
1	0	1	1	1	4
1	0	1	1	1	4
1	0	1	1	1	4
1	0	1	1	1	4
1	0	1	1	1	4
1	0	2	2	1	1
1	0	2	2	1	1
1	0	2	2	1	1
1	0	2	2	1	1
1	0	2	2	1	1
1	0	2	2	1	1
1	0	2	2	1	1
1	0	2	2	1	1
1	0	2	2	1	1
1	0	2	2	1	1
1	0	2	2	1	1
1	0	2	2	1	1
1	0	2	2	1	1
1	0	2	2	1	1
1	0	2	2	1	1
1	0	2	2	1	1
1	0	2	2	1	1
1	0	2	2	1	1
1	0	2	2	1	1

1	0	2	2	1	1
0	1	1	1	1	1
0	1	1	1	1	1
0	1	1	1	1	1
0	1	1	1	1	1
0	1	1	1	1	1
0	1	1	1	1	1
0	1	1	1	1	1
0	1	1	1	1	1
1	0	1	2	1	1
1	0	1	2	1	1
1	0	1	2	1	1
1	0	1	2	1	1
1	0	1	2	1	1
1	1	4	7	7	5
1	1	4	7	7	5
1	1	4	7	7	5
1	1	4	7	7	5
1	1	4	7	7	5
1	0	1	1	1	1
1	0	1	1	1	1
1	0	1	1	1	1
1	0	1	1	1	1
1	0	1	1	1	1
1	0	1	1	1	1
1	0	1	1	1	1
1	0	1	1	1	1
1	0	1	1	1	1
1	0	1	1	1	1
1	0	1	1	1	1
0	1	1	2	2	1
0	1	1	2	2	1
0	1	1	2	2	1
0	1	1	2	2	1
0	1	1	2	2	1
0	1	1	2	2	1
0	1	1	2	2	1
0	1	1	2	2	1
0	1	1	2	2	1
0	1	1	2	2	1
0	1	1	2	2	1
0	1	1	1	1	1
0	1	1	1	1	1
0	1	1	1	1	1
0	1	1	1	1	1
0	1	1	1	1	1
0	1	1	1	1	1
0	1	1	1	1	1
0	1	1	1	1	1
0	1	1	1	1	1

0	1	1	1	1	1
0	1	1	1	1	1
0	1	1	1	1	1
0	1	1	1	1	1
0	1	1	1	1	1
0	1	1	1	1	1
0	1	1	1	1	1
0	1	1	1	1	1
0	1	1	1	1	1
0	1	1	1	1	1
0	1	1	1	1	1
0	1	1	1	1	1
1	1	1	3	2	1
1	1	1	3	2	1
1	1	1	3	2	1
1	1	1	3	2	1
1	1	1	3	2	1
1	1	1	3	2	1
1	1	1	3	2	1
1	1	1	3	2	1

bm_cardiovasc	bm_meforme	bm_diabete	bm_hyperglycemie	bm_sante	bm_climat
4	2	1	1	4	4
4	2	1	1	4	4
4	2	1	1	4	4
4	2	1	1	4	4
2	1	1	1	3	3
2	1	1	1	3	3
2	1	1	1	3	3
1	3	1	1	5	3
1	3	1	1	5	3
1	3	1	1	5	3
1	3	1	1	5	3
1	3	1	1	5	3
1	3	1	1	5	3
1	3	1	1	5	3
6	6	2	2	5	1
6	6	2	2	5	1
6	6	2	2	5	1
6	6	2	2	5	1
6	6	2	2	5	1
6	6	2	2	5	1
6	6	2	2	5	1
6	6	2	2	5	1
6	6	2	2	5	1
6	6	2	2	5	1
6	6	2	2	5	1
6	6	2	2	5	1
6	6	2	2	5	1
6	6	2	2	5	1
6	6	2	2	5	1
6	6	2	2	5	1
6	6	2	2	5	1
6	6	2	2	5	1
6	6	2	2	5	1
6	6	2	2	5	1
3	3	1	1	1	1
6	1	6	6	7	7
3	3	1	1	6	3
3	3	1	1	6	3
3	3	1	1	6	3
3	3	1	1	6	3
3	3	1	1	6	3
3	2	1	1	5	1
3	2	1	1	5	1
3	2	1	1	5	1
3	2	1	1	5	1
4	5	2	2	4	3
4	5	2	2	4	3
4	5	2	2	4	3
4	5	2	2	4	3

4	5	2	2	4	3
4	5	2	2	4	3
4	5	2	2	4	3
4	5	2	2	4	3
4	5	2	2	4	3
4	5	2	2	4	3
1	1	1	1	4	3
3	3	1	1	4	4
3	3	1	1	4	4
3	3	1	1	4	4
3	3	1	1	4	4
3	3	1	1	4	4
4	3	1	1	4	3
4	3	1	1	4	3
4	3	1	1	4	3
4	3	1	1	4	3
4	3	1	1	4	3
4	3	1	1	4	3
4	3	1	1	4	3
4	3	1	1	4	3
4	3	1	1	4	3
4	3	1	1	4	3
4	3	1	1	4	3
4	3	1	1	4	3
4	3	1	1	4	3
4	3	1	1	4	3
4	3	1	1	4	3
4	3	1	1	4	3
4	3	1	1	4	3
4	3	1	1	4	3
4	3	1	1	4	3
1	1	1	1	1	1
1	1	1	1	1	1
1	1	1	1	1	1
1	1	1	1	1	1
1	1	1	1	1	1
1	1	1	1	1	1
1	1	1	1	1	1
1	1	1	1	1	1
1	1	1	1	1	1
1	1	1	1	1	1
1	1	1	1	1	1
1	1	1	1	1	1
1	1	1	1	1	1
1	1	1	1	1	1
1	1	1	1	1	1
2	2	1	1	3	3
2	2	1	1	3	3
2	2	1	1	3	3

5	5	2	2	3	2
5	5	2	2	3	2
3	5	1	1	7	4
3	5	1	1	7	4
3	5	1	1	7	4
3	5	1	1	7	4
3	5	1	1	7	4
3	5	1	1	7	4
3	5	1	1	7	4
1	2	1	1	3	4
1	2	1	1	3	4
1	2	1	1	3	4
1	2	1	1	3	4
1	2	1	1	3	4
1	1	1	1	4	1
3	2	1	1	5	5
3	2	1	1	5	5
3	2	1	1	5	5
2	4	1	1	6	2
2	4	1	1	6	2
2	4	1	1	6	2
2	4	1	1	6	2
2	4	1	1	6	2
2	3	1	1	4	2
2	3	1	1	4	2
2	3	1	1	4	2
2	3	1	1	4	2
2	3	1	1	4	2
5	6	1	1	4	7
5	6	1	1	4	7
5	6	1	1	4	7
5	6	1	1	4	7
5	6	1	1	4	7
5	6	1	1	4	7
5	6	1	1	4	7
5	6	1	1	4	7
5	6	1	1	4	7
5	6	1	1	4	7
5	6	1	1	4	7
5	6	1	1	4	7
5	6	1	1	4	7
5	6	1	1	4	7
5	6	1	1	4	7
5	6	1	1	4	7
5	6	1	1	4	7
5	6	1	1	4	7
5	6	1	1	4	7
5	6	1	1	4	7
1	4	1	1	5	1
1	4	1	1	5	1

1	4	1	1	5	1
1	4	1	1	5	1
1	4	1	1	5	1
1	4	1	1	5	1
1	4	1	1	5	1
1	4	1	1	5	1
1	4	1	1	5	1
1	4	1	1	5	1
1	4	1	1	5	1
1	4	1	1	5	1
1	4	1	1	5	1
1	4	1	1	5	1
1	4	1	1	5	1
1	2	1	1	2	2
1	2	1	1	2	2
2	1	1	2	3	2
2	1	1	2	3	2
2	1	1	2	3	2
2	1	1	2	3	2
2	1	1	2	3	2
2	1	1	2	3	2
2	1	1	2	3	2
2	1	1	2	3	2
2	1	1	2	3	2
2	1	1	2	3	2
2	1	1	2	3	2
6	4	3	1	4	1
6	4	3	1	4	1
6	4	3	1	4	1
6	4	3	1	4	1
6	4	3	1	4	1
6	4	3	1	4	1
4	5	2	2	4	3
4	5	2	2	4	3
4	5	2	2	4	3
4	5	2	2	4	3
4	5	2	2	4	3
4	5	2	2	4	3
1	1	1	1	4	4
1	1	1	1	4	4
1	1	1	1	4	4
1	1	1	1	4	4
1	1	1	1	4	4
4	6	1	1	6	4
4	6	1	1	6	4
4	6	1	1	6	4
4	1	1	1	4	2
4	1	1	1	4	2
4	1	1	1	4	2
4	1	1	1	4	2

4	1	1	1	4	2
4	1	1	1	4	2
4	1	1	1	4	2
4	3	1	1	5	3
4	3	1	1	5	3
4	3	1	1	5	3
4	3	1	1	5	3
4	3	1	1	5	3
2	6	1	1	5	1
2	6	1	1	5	1
2	2	1	1	6	3
2	2	1	1	6	3
2	2	1	1	6	3
2	2	1	1	6	3
2	2	1	1	6	3
2	2	1	1	6	3
2	2	1	1	6	3
2	2	1	1	6	3
2	2	1	1	6	3
2	2	1	1	6	3
2	2	1	1	6	3
2	2	1	1	6	3
2	2	1	1	6	3
2	2	1	1	6	3
2	2	1	1	6	3
2	2	1	1	6	3
2	2	1	1	6	3
2	2	1	1	6	3
2	2	1	1	6	3
2	2	1	1	6	3
3	7	1	1	5	6
3	7	1	1	5	6
3	7	1	1	5	6
3	7	1	1	5	6
3	7	1	1	5	6
3	7	1	1	5	6
2	2	1	1	2	2
2	2	1	1	2	2
2	2	1	1	2	2
2	2	1	1	2	2
2	2	1	1	2	2
2	2	1	1	2	2
2	2	1	1	2	2
2	2	1	1	2	2
2	2	1	1	2	2
1	5	2	1	5	5
1	5	2	1	5	5
1	5	2	1	5	5
1	5	2	1	5	5
1	5	2	1	5	5

1	5	2	1	5	5
1	5	2	1	5	5
1	5	2	1	5	5
1	5	2	1	5	5
1	5	2	1	5	5
1	5	2	1	5	5
1	5	2	1	5	5
1	5	2	1	5	5
1	5	2	1	5	5
1	5	2	1	5	5
4	4	2	1	5	1
4	4	2	1	5	1
4	4	2	1	5	1
4	4	2	1	5	1
4	4	2	1	5	1
2	2	2	2	2	2
2	2	2	2	2	2
2	2	2	2	2	2
2	2	2	2	2	2
4	4	1	1	7	6
4	4	1	1	7	6
4	4	1	1	7	6
4	4	1	1	7	6
4	4	1	1	7	6
4	4	1	1	7	6
4	4	1	1	7	6
4	4	1	1	7	6
4	4	1	1	7	6
4	4	1	1	7	6
4	4	1	1	7	6
4	4	1	1	7	6
3	1	1	1	4	1
3	1	1	1	4	1
3	1	1	1	4	1
3	1	1	1	4	1
3	1	1	1	4	1
3	1	1	1	4	1
3	1	1	1	4	1
3	1	1	1	4	1
3	1	1	1	4	1
3	1	1	1	4	1
3	1	1	1	4	1
3	1	1	1	4	1
3	1	1	1	4	1
3	1	1	1	4	1
3	1	1	1	4	1
3	1	1	1	4	1
3	1	1	1	4	1
3	1	1	1	4	1
3	1	1	1	4	1

3	1	1	1	4	1
1	1	1	1	1	1
1	1	1	1	1	1
1	1	1	1	1	1
1	1	1	1	1	1
1	1	1	1	1	1
1	1	1	1	1	1
1	1	1	1	1	1
1	1	1	1	1	1
1	2	1	1	3	1
1	2	1	1	3	1
1	2	1	1	3	1
1	2	1	1	3	1
1	2	1	1	3	1
6	3	3	1	6	5
6	3	3	1	6	5
6	3	3	1	6	5
6	3	3	1	6	5
6	3	3	1	6	5
1	1	1	1	7	1
1	1	1	1	7	1
1	1	1	1	7	1
1	1	1	1	7	1
1	1	1	1	7	1
1	1	1	1	7	1
1	1	1	1	7	1
1	1	1	1	7	1
1	1	1	1	7	1
1	1	1	1	7	1
1	1	1	1	7	1
5	2	1	1	6	4
5	2	1	1	6	4
5	2	1	1	6	4
5	2	1	1	6	4
5	2	1	1	6	4
5	2	1	1	6	4
5	2	1	1	6	4
5	2	1	1	6	4
5	2	1	1	6	4
5	2	1	1	6	4
5	2	1	1	6	4
3	3	1	1	6	3
3	3	1	1	6	3
7	6	1	1	7	4
7	6	1	1	7	4
7	6	1	1	7	4
7	6	1	1	7	4
7	6	1	1	7	4
7	6	1	1	7	4
7	6	1	1	7	4

7	6	1	1	7	4
7	6	1	1	7	4
7	6	1	1	7	4
7	6	1	1	7	4
7	6	1	1	7	4
7	6	1	1	7	4
7	6	1	1	7	4
7	6	1	1	7	4
7	6	1	1	7	4
7	6	1	1	7	4
7	6	1	1	7	4
7	6	1	1	7	4
4	5	2	1	4	3
4	5	2	1	4	3
4	5	2	1	4	3
4	5	2	1	4	3
4	5	2	1	4	3
4	5	2	1	4	3
4	5	2	1	4	3
4	5	2	1	4	3

bm_horaire	bapad_med	bm	rhd_priomed	ado1_priomed	ado2_priomed	priomed
1	25	2,27	1	1	0	110
1	25	2,27	1	1	0	110
1	25	2,27	1	1	0	110
1	25	2,27	1	1	0	110
1	16	1,45	0	1	0	10
1	16	1,45	0	1	0	10
1	16	1,45	0	1	0	10
2	20	1,82	1	0	0	100
2	20	1,82	1	0	0	100
2	20	1,82	1	0	0	100
2	20	1,82	1	0	0	100
2	20	1,82	1	0	0	100
2	20	1,82	1	0	0	100
2	20	1,82	1	0	0	100
1	37	3,36	0	1	0	10
1	37	3,36	0	1	0	10
1	37	3,36	0	1	0	10
1	37	3,36	0	1	0	10
1	37	3,36	0	1	0	10
1	37	3,36	0	1	0	10
1	37	3,36	0	1	0	10
1	37	3,36	0	1	0	10
1	37	3,36	0	1	0	10
1	37	3,36	0	1	0	10
1	37	3,36	0	1	0	10
1	37	3,36	0	1	0	10
1	37	3,36	0	1	0	10
1	37	3,36	0	1	0	10
1	37	3,36	0	1	0	10
1	37	3,36	0	1	0	10
1	37	3,36	0	1	0	10
1	37	3,36	0	1	0	10
1	37	3,36	0	1	0	10
1	37	3,36	0	1	0	10
1	17	1,55	1	0	0	100
7	58	5,27	1	0	0	100
3	26	2,36	1	0	0	100
3	26	2,36	1	0	0	100
3	26	2,36	1	0	0	100
3	26	2,36	1	0	0	100
3	26	2,36	1	0	0	100
3	24	2,18	1	0	0	100
3	24	2,18	1	0	0	100
3	24	2,18	1	0	0	100
3	24	2,18	1	0	0	100
4	32	2,91	1	1	0	110
4	32	2,91	1	1	0	110
4	32	2,91	1	1	0	110
4	32	2,91	1	1	0	110

4	32	2,91	1	1	0	110
4	32	2,91	1	1	0	110
4	32	2,91	1	1	0	110
4	32	2,91	1	1	0	110
4	32	2,91	1	1	0	110
4	32	2,91	1	1	0	110
3	18	1,64	1	1	0	110
4	29	2,64	0	0	1	1
4	29	2,64	0	0	1	1
4	29	2,64	0	0	1	1
4	29	2,64	0	0	1	1
4	29	2,64	0	0	1	1
6	31	2,82	1	0	0	100
6	31	2,82	1	0	0	100
6	31	2,82	1	0	0	100
6	31	2,82	1	0	0	100
6	31	2,82	1	0	0	100
6	31	2,82	1	0	0	100
6	31	2,82	1	0	0	100
6	31	2,82	1	0	0	100
6	31	2,82	1	0	0	100
6	31	2,82	1	0	0	100
6	31	2,82	1	0	0	100
6	31	2,82	1	0	0	100
6	31	2,82	1	0	0	100
6	31	2,82	1	0	0	100
6	31	2,82	1	0	0	100
6	31	2,82	1	0	0	100
6	31	2,82	1	0	0	100
6	31	2,82	1	0	0	100
6	31	2,82	1	0	0	100
6	31	2,82	1	0	0	100
1	11	1	1	0	0	100
1	11	1	1	0	0	100
1	11	1	1	0	0	100
1	11	1	1	0	0	100
1	11	1	1	0	0	100
1	11	1	1	0	0	100
1	11	1	1	0	0	100
1	11	1	1	0	0	100
1	11	1	1	0	0	100
1	11	1	1	0	0	100
1	11	1	1	0	0	100
1	11	1	1	0	0	100
1	11	1	1	0	0	100
1	11	1	1	0	0	100
1	11	1	1	0	0	100
5	23	2,09	1	0	0	100
5	23	2,09	1	0	0	100
5	23	2,09	1	0	0	100

2	37	3,36	1	1	0	110
2	37	3,36	1	1	0	110
1	28	2,55	1	1	0	110
1	28	2,55	1	1	0	110
1	28	2,55	1	1	0	110
1	28	2,55	1	1	0	110
1	28	2,55	1	1	0	110
1	28	2,55	1	1	0	110
1	28	2,55	1	1	0	110
3	21	1,91	1	0	0	100
3	21	1,91	1	0	0	100
3	21	1,91	1	0	0	100
3	21	1,91	1	0	0	100
3	21	1,91	1	0	0	100
1	14	1,27	1	0	0	100
1	24	2,18	1	0	0	100
1	24	2,18	1	0	0	100
1	24	2,18	1	0	0	100
3	25	2,27	1	0	0	100
3	25	2,27	1	0	0	100
3	25	2,27	1	0	0	100
3	25	2,27	1	0	0	100
3	25	2,27	1	0	0	100
3	23	2,09	1	0	0	100
3	23	2,09	1	0	0	100
3	23	2,09	1	0	0	100
3	23	2,09	1	0	0	100
3	23	2,09	1	0	0	100
7	44	4	1	0	0	100
7	44	4	1	0	0	100
7	44	4	1	0	0	100
7	44	4	1	0	0	100
7	44	4	1	0	0	100
7	44	4	1	0	0	100
7	44	4	1	0	0	100
7	44	4	1	0	0	100
7	44	4	1	0	0	100
7	44	4	1	0	0	100
7	44	4	1	0	0	100
7	44	4	1	0	0	100
7	44	4	1	0	0	100
7	44	4	1	0	0	100
7	44	4	1	0	0	100
7	44	4	1	0	0	100
7	44	4	1	0	0	100
7	44	4	1	0	0	100
7	44	4	1	0	0	100
7	44	4	1	0	0	100
4	27	2,45	1	0	0	100
4	27	2,45	1	0	0	100

4	27	2,45	1	0	0	100
4	27	2,45	1	0	0	100
4	27	2,45	1	0	0	100
4	27	2,45	1	0	0	100
4	27	2,45	1	0	0	100
4	27	2,45	1	0	0	100
4	27	2,45	1	0	0	100
4	27	2,45	1	0	0	100
4	27	2,45	1	0	0	100
4	27	2,45	1	0	0	100
4	27	2,45	1	0	0	100
4	27	2,45	1	0	0	100
4	27	2,45	1	0	0	100
1	14	1,27	1	0	0	100
1	14	1,27	1	0	0	100
3	25	2,27	1	0	0	100
3	25	2,27	1	0	0	100
3	25	2,27	1	0	0	100
3	25	2,27	1	0	0	100
3	25	2,27	1	0	0	100
3	25	2,27	1	0	0	100
3	25	2,27	1	0	0	100
3	25	2,27	1	0	0	100
3	25	2,27	1	0	0	100
3	25	2,27	1	0	0	100
3	25	2,27	1	0	0	100
3	29	2,64	1	0	0	100
3	29	2,64	1	0	0	100
3	29	2,64	1	0	0	100
3	29	2,64	1	0	0	100
3	29	2,64	1	0	0	100
3	29	2,64	1	0	0	100
1	28	2,55	1	0	0	100
1	28	2,55	1	0	0	100
1	28	2,55	1	0	0	100
1	28	2,55	1	0	0	100
1	28	2,55	1	0	0	100
1	28	2,55	1	0	0	100
4	21	1,91	1	1	0	110
4	21	1,91	1	1	0	110
4	21	1,91	1	1	0	110
4	21	1,91	1	1	0	110
4	21	1,91	1	1	0	110
4	37	3,36	1	0	0	100
4	37	3,36	1	0	0	100
4	37	3,36	1	0	0	100
1	19	1,73	1	1	0	110
1	19	1,73	1	1	0	110
1	19	1,73	1	1	0	110
1	19	1,73	1	1	0	110

1	19	1,73	1	1	0	110
1	19	1,73	1	1	0	110
1	19	1,73	1	1	0	110
5	29	2,64	1	0	0	100
5	29	2,64	1	0	0	100
5	29	2,64	1	0	0	100
5	29	2,64	1	0	0	100
5	29	2,64	1	0	0	100
4	27	2,45	1	1	0	110
4	27	2,45	1	1	0	110
2	23	2,09	1	0	0	100
2	23	2,09	1	0	0	100
2	23	2,09	1	0	0	100
2	23	2,09	1	0	0	100
2	23	2,09	1	0	0	100
2	23	2,09	1	0	0	100
2	23	2,09	1	0	0	100
2	23	2,09	1	0	0	100
2	23	2,09	1	0	0	100
2	23	2,09	1	0	0	100
2	23	2,09	1	0	0	100
2	23	2,09	1	0	0	100
2	23	2,09	1	0	0	100
2	23	2,09	1	0	0	100
2	23	2,09	1	0	0	100
2	23	2,09	1	0	0	100
2	23	2,09	1	0	0	100
2	23	2,09	1	0	0	100
2	23	2,09	1	0	0	100
2	23	2,09	1	0	0	100
6	33	3	1	0	0	100
6	33	3	1	0	0	100
6	33	3	1	0	0	100
6	33	3	1	0	0	100
6	33	3	1	0	0	100
6	33	3	1	0	0	100
1	16	1,45	1	0	0	100
1	16	1,45	1	0	0	100
1	16	1,45	1	0	0	100
1	16	1,45	1	0	0	100
1	16	1,45	1	0	0	100
1	16	1,45	1	0	0	100
1	16	1,45	1	0	0	100
1	16	1,45	1	0	0	100
1	16	1,45	1	0	0	100
5	39	3,55	1	0	0	100
5	39	3,55	1	0	0	100
5	39	3,55	1	0	0	100
5	39	3,55	1	0	0	100
5	39	3,55	1	0	0	100

5	39	3,55	1	0	0	100
5	39	3,55	1	0	0	100
5	39	3,55	1	0	0	100
5	39	3,55	1	0	0	100
5	39	3,55	1	0	0	100
5	39	3,55	1	0	0	100
5	39	3,55	1	0	0	100
5	39	3,55	1	0	0	100
5	39	3,55	1	0	0	100
5	39	3,55	1	0	0	100
1	27	2,45	1	0	0	100
1	27	2,45	1	0	0	100
1	27	2,45	1	0	0	100
1	27	2,45	1	0	0	100
1	27	2,45	1	0	0	100
2	22	2	1	0	0	100
2	22	2	1	0	0	100
2	22	2	1	0	0	100
2	22	2	1	0	0	100
2	32	2,91	1	0	0	100
2	32	2,91	1	0	0	100
2	32	2,91	1	0	0	100
2	32	2,91	1	0	0	100
2	32	2,91	1	0	0	100
2	32	2,91	1	0	0	100
2	32	2,91	1	0	0	100
2	32	2,91	1	0	0	100
2	32	2,91	1	0	0	100
2	32	2,91	1	0	0	100
2	32	2,91	1	0	0	100
2	32	2,91	1	0	0	100
1	18	1,64	1	0	0	100
1	18	1,64	1	0	0	100
1	18	1,64	1	0	0	100
1	18	1,64	1	0	0	100
1	18	1,64	1	0	0	100
1	18	1,64	1	0	0	100
1	18	1,64	1	0	0	100
1	18	1,64	1	0	0	100
1	18	1,64	1	0	0	100
1	18	1,64	1	0	0	100
1	18	1,64	1	0	0	100
1	18	1,64	1	0	0	100
1	18	1,64	1	0	0	100
1	18	1,64	1	0	0	100
1	18	1,64	1	0	0	100
1	18	1,64	1	0	0	100
1	18	1,64	1	0	0	100
1	18	1,64	1	0	0	100
1	18	1,64	1	0	0	100

1	18	1,64	1	0	0	100
1	11	1	1	0	0	100
1	11	1	1	0	0	100
1	11	1	1	0	0	100
1	11	1	1	0	0	100
1	11	1	1	0	0	100
1	11	1	1	0	0	100
1	11	1	1	0	0	100
1	11	1	1	0	0	100
1	15	1,36	1	0	0	100
1	15	1,36	1	0	0	100
1	15	1,36	1	0	0	100
1	15	1,36	1	0	0	100
1	15	1,36	1	0	0	100
5	52	4,73	1	0	0	100
5	52	4,73	1	0	0	100
5	52	4,73	1	0	0	100
5	52	4,73	1	0	0	100
5	52	4,73	1	0	0	100
2	18	1,64	0	0	1	1
2	18	1,64	0	0	1	1
2	18	1,64	0	0	1	1
2	18	1,64	0	0	1	1
2	18	1,64	0	0	1	1
2	18	1,64	0	0	1	1
2	18	1,64	0	0	1	1
2	18	1,64	0	0	1	1
2	18	1,64	0	0	1	1
2	18	1,64	0	0	1	1
2	18	1,64	0	0	1	1
2	27	2,45	1	0	0	100
2	27	2,45	1	0	0	100
2	27	2,45	1	0	0	100
2	27	2,45	1	0	0	100
2	27	2,45	1	0	0	100
2	27	2,45	1	0	0	100
2	27	2,45	1	0	0	100
2	27	2,45	1	0	0	100
2	27	2,45	1	0	0	100
2	27	2,45	1	0	0	100
2	27	2,45	1	0	0	100
4	25	2,27	0	1	0	10
4	25	2,27	0	1	0	10
6	36	3,27	1	0	0	100
6	36	3,27	1	0	0	100
6	36	3,27	1	0	0	100
6	36	3,27	1	0	0	100
6	36	3,27	1	0	0	100
6	36	3,27	1	0	0	100
6	36	3,27	1	0	0	100

6	36	3,27	1	0	0	100
6	36	3,27	1	0	0	100
6	36	3,27	1	0	0	100
6	36	3,27	1	0	0	100
6	36	3,27	1	0	0	100
6	36	3,27	1	0	0	100
6	36	3,27	1	0	0	100
6	36	3,27	1	0	0	100
6	36	3,27	1	0	0	100
6	36	3,27	1	0	0	100
6	36	3,27	1	0	0	100
6	36	3,27	1	0	0	100
5	31	2,82	1	1	0	110
5	31	2,82	1	1	0	110
5	31	2,82	1	1	0	110
5	31	2,82	1	1	0	110
5	31	2,82	1	1	0	110
5	31	2,82	1	1	0	110
5	31	2,82	1	1	0	110
5	31	2,82	1	1	0	110

fuzzyfumeur	fumeur	patient	datapatients	male_pat	age_pat	duree_diab	hba1c_known
NA	NA	60	1	0	71	40	1
NA	NA	61	1	1	70	12	1
NA	NA	62	1	0	59	4	1
NA	NA	63	1	1	54	9	1
NA	NA	64	1	0	74	15	1
NA	NA	65	1	0	80	18	0
NA	NA	66	1	1	71	10	1
NA	NA	67	1	1	62	10	1
NA	NA	68	1	0	63	3	1
NA	NA	69	1	1	69	10	1
NA	NA	70	1	0	69	10	1
NA	NA	71	1	1	73	2	1
NA	NA	72	1	0	77	15	1
NA	NA	73	1	1	58	5	1
NA	NA	74	1	0	80	15	0
NA	NA	75	1	1	54	2	1
NA	NA	76	1	0	71	8	0
NA	NA	77	1	1	77	15	1
NA	NA	78	1	1	69	2	1
NA	NA	79	1	1	64	12	1
NA	NA	80	1	1	70	10	1
NA	NA	81	1	1	66	20	1
NA	NA	82	1	1	61	6	1
NA	NA	83	1	0	69	3	1
NA	NA	84	1	1	80	14	0
NA	NA	85	1	0	78	5	1
NA	NA	86	1	1	59	5	1
NA	NA	87	1	0	81	12	1
NA	NA	88	1	0	44	3	1
NA	NA	89	1	1	69	4	1
NA	NA	90	1	0	61	18	1
NA	NA	91	1	0	70	8	0
NA	NA	92	1	0	68	14	1
NA	NA	93	1	0	30	3	1
NA	NA	1	1	1	59	14	0
NA	NA	2	1	0	67	4	1
NA	NA	40	1	1	65	10	0
NA	NA	41	1	0	70	4	1
NA	NA	42	1	0	70	8	1
NA	NA	43	1	0	73	10	0
NA	NA	44	1	1	62	5	1
NA	NA	45	1	0	73	5	1
NA	NA	46	1	1	81	6	1
NA	NA	47	1	1	55	3	0
NA	NA	48	1	0	61	3	1
NA	NA	49	1	0	48	11	1
NA	NA	50	1	1	63	6	1
NA	NA	51	1	1	53	7	0
NA	NA	52	1	1	54	5	1

NA	NA	53	1	0	48	12	0
NA	NA	54	1	1	60	14	0
NA	NA	55	1	1	56	7	1
NA	NA	56	1	0	47	2	1
NA	NA	57	1	0	55	8	1
NA	NA	58	1	0	50	7	0
NA	NA	59	1	1	75	9	1
NA	NA	94	1	1	74	5	1
NA	NA	95	1	0	68	1	0
NA	NA	96	1	0	57	1	0
NA	NA	97	1	0	69	2	0
NA	NA	98	1	0	63	2	1
NA	NA	99	1	1	60	10	1
NA	NA	100	1	0	75	20	0
NA	NA	101	1	0	65	30	1
NA	NA	102	1	1	79	25	0
NA	NA	103	1	1	70	30	0
NA	NA	104	1	0	20	15	1
NA	NA	105	1	0	61	10	1
NA	NA	106	1	1	50	8	1
NA	NA	107	1	1	56	10	0
NA	NA	108	1	1	48	3	0
NA	NA	109	1	1	69	15	1
NA	NA	110	1	1	57	10	1
NA	NA	111	1	1	68	20	0
NA	NA	112	1	0	76	15	0
NA	NA	113	1	0	43	2	0
NA	NA	114	1	0	57	8	1
NA	NA	115	1	1	55	9	1
NA	NA	116	1	0	65	18	0
NA	NA	117	1	1	68	20	0
NA	NA	118	1	1	49	7,58	0
NA	NA	119	1	1	74	4	1
NA	NA	120	1	0	75	12	1
NA	NA	121	1	0	64	3	1
NA	NA	122	1	0	52	1	1
NA	NA	123	1	1	47	8	1
NA	NA	124	1	1	63	8	1
NA	NA	125	1	0	63	11	0
NA	NA	126	1	1	62	10	0
NA	NA	127	1	1	59	13	1
NA	NA	128	1	1	67	16	1
NA	NA	129	1	1	72	2	1
NA	NA	130	1	0	73	5	1
NA	NA	131	1	1	69	2	1
NA	NA	132	1	1	68	10	1
NA	NA	133	1	0	79	4	1
NA	NA	140	1	0	66	7	0
NA	NA	141	1	0	85	1	0
NA	NA	142	1	1	64	7	1

NA	NA	143	1	1	71	20	0
NA	NA	144	1	0	66	2	1
NA	NA	145	1	1	71	9,64	1
NA	NA	146	1	1	60	2	1
NA	NA	147	1	1	55	8	1
NA	NA	148	1	0	93	1	0
NA	NA	149	1	1	58	4	1
NA	NA	150	1	1	47	2	0
NA	NA	151	1	1	66	10	1
NA	NA	176	1	1	59	10	0
NA	NA	177	1	0	79	35	0
NA	NA	178	1	1	68	8	1
NA	NA	179	1	0	57	9	0
NA	NA	180	1	0	49	6	1
NA	NA	181	1	0	64	5	1
NA	NA	182	1	0	69	30	1
NA	NA	183	1	1	60	30	1
NA	NA	184	1	0	68	10	0
NA	NA	193	1	1	60	1	1
NA	NA	194	1	1	50	3	1
NA	NA	195	1	1	62	4	1
NA	NA	196	1	0	75	13,69	1
NA	NA	197	1	1	61	15	1
NA	NA	198	1	0	59	5	1
NA	NA	199	1	1	59	7	1
NA	NA	200	1	1	60	3	1
NA	NA	201	1	0	59	2	1
NA	NA	202	1	1	57	4	1
NA	NA	203	1	1	88	20	0
NA	NA	204	1	1	75	5	0
NA	NA	205	1	0	75	20	1
NA	NA	206	1	1	53	7	1
NA	NA	207	1	0	56	10	0
NA	NA	208	1	1	62	10	1
NA	NA	209	1	0	57	2	1
NA	NA	210	1	1	61	3	0
NA	NA	211	1	1	61	5	0
NA	NA	212	1	1	72	5	1
NA	NA	213	1	1	70	2	0
NA	NA	214	1	1	80	10	1
NA	NA	215	1	1	75	2	0
NA	NA	216	1	1	56	2	0
NA	NA	217	1	1	63	12	1
NA	NA	218	1	1	76	20	0
NA	NA	219	1	1	75	3	0
NA	NA	220	1	1	58	13	1
NA	NA	221	1	1	53	10	1
NA	NA	222	1	0	54	5	0
NA	NA	239	1	0	58	6	0
NA	NA	240	1	0	57	10	1

NA	NA	241	1	1	63	7	0
NA	NA	242	1	1	60	9	1
NA	NA	243	1	0	48	3	0
NA	NA	244	1	1	53	8	1
NA	NA	245	1	0	56	3	0
NA	NA	246	1	0	48	10	0
NA	NA	247	1	0	61	10	0
NA	NA	248	1	1	60	3	1
NA	NA	249	1	0	47	2	1
NA	NA	250	1	1	57	15	0
NA	NA	251	1	1	50	5	0
NA	NA	252	1	1	68	12	0
NA	NA	253	1	0	58	15	0
NA	NA	254	1	1	61	5	1
NA	NA	255	1	0	76	16	1
NA	NA	256	1	1	63	15	1
NA	NA	257	1	1	60	30	1
NA	NA	258	1	1	56	1	1
NA	NA	259	1	1	49	6	0
NA	NA	260	1	1	75	20	1
NA	NA	261	1	0	77	1	1
NA	NA	262	1	0	73	16	1
NA	NA	263	1	1	61	2	1
NA	NA	264	1	0	61	2	1
NA	NA	265	1	0	61	4	1
NA	NA	266	1	1	60	2	0
NA	NA	295	1	1	65	20	1
NA	NA	296	1	0	76	15	1
NA	NA	297	1	0	72	6	1
NA	NA	298	1	0	67	20	1
NA	NA	299	1	1	73	4	1
NA	NA	300	1	1	69	25	1
NA	NA	301	1	1	53	5	1
NA	NA	302	1	1	56	6	1
NA	NA	303	1	1	61	2	1
NA	NA	304	1	1	86	10	0
NA	NA	305	1	1	48	5	0
NA	NA	306	1	0	79	5	1
NA	NA	339	1	0	73	10	1
NA	NA	340	1	1	60	5	1
NA	NA	341	1	1	44	3	1
NA	NA	342	1	0	64	3	1
NA	NA	343	1	1	57	2	1
NA	NA	3	1	0	47	4	0
NA	NA	4	1	1	69	4	1
NA	NA	5	1	1	69	10	1
NA	NA	6	1	1	73	4	0
NA	NA	7	1	0	74	10	1
NA	NA	8	1	0	60	7	1
NA	NA	9	1	1	63	2	1

NA	NA	10	1	0	70	20	1
NA	NA	11	1	1	56	20	1
NA	NA	12	1	0	83	7	1
NA	NA	13	1	1	72	15	1
NA	NA	14	1	1	57	10	1
NA	NA	15	1	1	50	2	0
NA	NA	16	1	1	71	20	1
NA	NA	17	1	0	60	25	1
NA	NA	18	1	1	59	0,5	1
NA	NA	19	1	0	72	10	1
NA	NA	20	1	0	80	10	0
NA	NA	21	1	0	99	3	0
NA	NA	22	1	1	77	25	1
NA	NA	23	1	0	75	5	1
NA	NA	24	1	1	78	29	0
NA	NA	25	1	1	85	15	0
NA	NA	26	1	1	72	27	0
NA	NA	27	1	1	63	18	0
NA	NA	28	1	1	77	13	1
NA	NA	29	1	0	88	7	0
NA	NA	30	1	1	83	3	0
NA	NA	31	1	0	63	6	1
NA	NA	32	1	0	70	2	0
NA	NA	33	1	0	78	9	0
NA	NA	34	1	0	85	21	0
NA	NA	35	1	1	75	15	1
NA	NA	36	1	0	38	1	0
NA	NA	37	1	1	61	5	0
NA	NA	38	1	0	63	2	0
NA	NA	39	1	1	82	13	0
NA	NA	134	1	1	72	8	1
NA	NA	135	1	0	65	40	1
NA	NA	136	1	1	85	4	0
NA	NA	137	1	0	55	4	1
NA	NA	138	1	0	69	20	1
NA	NA	139	1	1	59	5	0
NA	NA	152	1	1	50	8	1
NA	NA	153	1	0	61	15	1
NA	NA	154	1	1	70	20	1
NA	NA	155	1	0	85	30	1
NA	NA	156	1	1	49	1	1
NA	NA	157	1	1	41	3	1
NA	NA	158	1	1	59	4	1
NA	NA	159	1	0	71	15	1
NA	NA	160	1	1	67	10	1
NA	NA	161	1	1	72	10	1
NA	NA	162	1	1	58	15	1
NA	NA	163	1	1	59	3	1
NA	NA	164	1	1	53	1	0
NA	NA	165	1	1	77	10	1

NA	NA	166	1	1	72	7	1
NA	NA	167	1	1	56	2	1
NA	NA	168	1	1	59	10	1
NA	NA	169	1	0	35	1	1
NA	NA	170	1	1	68	6	1
NA	NA	171	1	1	73	3	1
NA	NA	172	1	0	50	15	1
NA	NA	173	1	0	63	20	1
NA	NA	174	1	1	54	10	1
NA	NA	175	1	1	52	3	1
NA	NA	188	1	0	78	33	1
NA	NA	189	1	0	54	5	1
NA	NA	190	1	1	58	7	1
NA	NA	191	1	0	67	4	1
NA	NA	192	1	1	57	1	1
NA	NA	223	1	0	56	4	1
NA	NA	224	1	1	58	10	1
NA	NA	225	1	1	57	4	1
NA	NA	226	1	0	58	10	1
NA	NA	227	1	0	72	3	1
NA	NA	228	1	0	50	4	1
NA	NA	229	1	1	67	6	0
NA	NA	230	1	1	76	10	0
NA	NA	231	1	0	66	3	0
NA	NA	232	1	0	70	6	1
NA	NA	233	1	0	73	10	1
NA	NA	234	1	1	57	2	0
NA	NA	235	1	1	63	4	1
NA	NA	236	1	0	72	12	0
NA	NA	237	1	0	78	20	0
NA	NA	238	1	0	80	30	0
NA	NA	267	1	1	69	6	0
NA	NA	268	1	1	76	7	0
NA	NA	269	1	0	81	5	0
NA	NA	270	1	1	61	14	1
NA	NA	271	1	1	70	8	0
NA	NA	272	1	1	64	3	0
NA	NA	273	1	1	64	3	0
NA	NA	274	1	1	62	2	1
NA	NA	275	1	1	63	9	1
NA	NA	276	1	1	68	7	0
NA	NA	277	1	0	55	1	1
NA	NA	278	1	0	58	4	0
NA	NA	279	1	1	65	9	0
NA	NA	280	1	1	77	8	1
NA	NA	281	1	1	74	21	0
NA	NA	282	1	1	60	2	0
NA	NA	283	1	1	89	3	0
NA	NA	284	1	1	65	20	1
NA	NA	285	1	1	58	2	1

NA	NA	286	1	0	67	10	1
NA	NA	287	1	1	63	13	0
NA	NA	288	1	1	53	0,04	0
NA	NA	289	1	1	57	8,81	0
NA	NA	290	1	0	79	12	0
NA	NA	291	1	1	72	2	0
NA	NA	292	1	1	70	15	1
NA	NA	293	1	1	54	10	0
NA	NA	294	1	1	58	10	0
NA	NA	307	1	1	71	14	0
NA	NA	308	1	1	72	6	0
NA	NA	309	1	1	57	6	1
NA	NA	310	1	1	61	11	1
NA	NA	311	1	0	70	12	1
NA	NA	312	1	1	76	10	1
NA	NA	313	1	1	71	1	0
NA	NA	314	1	1	72	4	0
NA	NA	315	1	0	71	5	1
NA	NA	316	1	0	70	6	1
NA	NA	317	1	1	69	9	1
NA	NA	318	1	0	64	6	1
NA	NA	319	1	0	74	4	1
NA	NA	320	1	1	63	12,46	1
NA	NA	321	1	0	77	7	1
NA	NA	322	1	0	83	19,41	1
NA	NA	323	1	1	80	13,68	1
NA	NA	324	1	1	74	17	1
NA	NA	325	1	1	63	8	1
NA	NA	326	1	0	71	24	1
NA	NA	327	1	1	72	12	1
NA	NA	328	1	0	71	35	0
NA	NA	329	1	1	77	15	0
NA	NA	330	1	0	74	25	1
NA	NA	331	1	0	70	12	1
NA	NA	332	1	0	74	18	0
NA	NA	333	1	1	65	2	0
NA	NA	334	1	1	68	13	0
NA	NA	335	1	1	58	20	0
NA	NA	336	1	1	60	15	0
NA	NA	337	1	0	78	3	1
NA	NA	338	1	0	76	4	1
NA	NA	344	1	1	79	29	1
NA	NA	345	1	1	71	10	1
NA	NA	346	1	1	60	20	1
NA	NA	347	1	1	69	20	1
NA	NA	348	1	1	59	7	1
NA	NA	349	1	1	58	8	1
NA	NA	350	1	0	67	9	1
NA	NA	351	1	0	57	3	0
NA	NA	352	1	0	71	31	1

NA	NA	353	1	1	49	8	0
NA	NA	354	1	0	53	0,75	0
NA	NA	355	1	0	46	13	0
NA	NA	356	1	1	59	5	1
NA	NA	357	1	0	69	19	1
NA	NA	358	1	0	81	20	1
NA	NA	359	1	1	72	26	1
NA	NA	360	1	1	58	6	1
NA	NA	361	1	1	61	14	1
NA	NA	362	1	1	62	19	1
NA	NA	363	1	0	75	14	1
NA	NA	364	1	1	65	25	1
NA	NA	365	1	1	56	10	1
NA	NA	366	1	1	61	12	1
NA	NA	367	1	0	48	6	1
NA	NA	368	1	1	66	10	1
NA	NA	369	1	1	58	5	1
NA	NA	370	1	0	60	12	1
NA	NA	371	1	1	59	6	1
NA	NA	372	1	0	63	2	1

hba1c_val	sport_pat	cyclisme	natation	marche	nb_sport	frq_sport	tm_sport	ordr_stoptbc
8,1	1	0	0	1	1	3	3	0
7,8	1	0	0	1	1	2	3	1
6,5	1	0	0	1	1	2	3	0
7,6	1	0	0	1	1	1	3	0
8,1	0	0	0	0	0	1	1	0
NA	0	0	0	0	0	1	1	0
6,7	1	0	0	1	1	1	3	1
6,3	0	0	0	0	0	1	1	0
6,3	1	0	0	1	1	2	2	0
6,6	1	0	0	1	1	3	3	1
6,3	0	0	0	0	0	1	1	0
7,4	1	0	0	1	1	2	2	1
7,6	0	0	0	0	0	1	1	0
6,6	1	0	0	1	1	3	3	0
NA	0	0	0	0	0	1	1	0
6,4	0	0	0	1	1	1	1	0
NA	0	0	0	0	0	1	1	0
7,1	1	0	0	1	1	3	2	1
6,9	1	0	0	1	1	3	2	0
8,1	0	0	0	0	0	1	1	1
6,3	0	0	0	0	0	1	1	0
6,3	1	0	0	1	1	3	3	1
5,9	1	0	0	0	0	3	3	1
5,9	0	0	0	0	0	1	1	0
NA	1	1	0	0	1	3	1	1
7	0	0	0	0	0	1	1	0
6,1	1	0	0	1	1	2	3	0
9,4	0	0	0	0	0	1	1	0
7,1	1	0	0	1	1	3	3	0
6,8	1	0	0	1	1	2	3	1
6,8	0	0	0	0	0	1	1	0
NA	0	0	0	0	0	1	1	0
7,1	0	0	0	1	1	1	1	0
5,9	1	0	1	1	2	3	3	0
NA	0	0	0	0	0	1	1	1
7,5	1	0	0	1	1	2	3	0
NA	1	0	0	1	1	3	3	1
7,5	1	0	0	1	1	2	2	1
8	0	0	0	1	1	1	1	0
NA	1	0	0	1	1	3	3	0
7,2	1	0	0	1	1	3	3	0
6,9	1	0	0	1	1	3	2	0
6,1	0	0	0	0	0	1	1	0
NA	0	0	0	1	1	2	1	1
7,1	1	0	0	1	1	2	2	0
7,6	0	0	0	0	0	1	1	1
8,2	0	0	0	0	0	1	1	1
NA	0	0	0	0	0	1	1	0
7,9	0	0	0	0	0	1	1	1

NA	0	0	0	1	1	1	1	0
NA	0	0	0	0	0	1	1	1
7,2	1	1	0	1	2	2	3	0
6,6	1	1	0	1	2	3	3	1
7,2	0	0	0	0	0	1	1	1
NA	0	0	0	1	1	1	1	0
7,4	1	1	0	0	1	1	3	1
6,8	0	0	0	1	1	2	1	0
NA	0	0	0	1	1	1	2	0
NA	1	1	1	1	3	2	3	0
NA	1	0	0	1	1	3	1	0
5,9	0	0	0	0	0	1	1	0
8,5	1	0	0	1	1	2	2	1
NA	0	0	0	0	0	1	1	0
8,9	0	0	0	0	0	1	1	1
NA	1	0	0	1	1	2	3	0
NA	1	0	0	1	1	2	3	0
6,7	0	0	1	0	1	1	2	0
6,5	1	1	0	1	2	2	2	0
7,7	0	0	0	0	0	1	1	1
NA	0	0	0	0	0	1	1	1
NA	0	0	0	0	0	1	1	1
5,9	1	0	0	1	1	3	3	1
7,8	0	0	0	1	1	2	1	0
NA	1	1	0	1	2	3	3	1
NA	0	0	0	0	0	1	1	0
NA	1	0	1	1	2	2	3	0
6,2	0	0	0	1	1	1	2	0
6,7	0	0	0	0	0	1	1	0
NA	0	0	0	0	0	1	1	0
NA	1	0	0	1	1	3	3	0
NA	1	0	0	0	0	1	3	1
7	1	1	0	1	2	3	3	0
8,1	0	0	0	1	1	1	1	0
5,9	0	0	0	0	0	1	1	0
6,1	0	1	0	1	2	1	1	0
9,1	1	0	0	1	1	3	2	1
8	0	0	0	0	0	1	1	1
NA	1	0	0	1	1	3	2	0
NA	0	0	0	0	0	1	1	1
6	0	1	0	1	2	1	1	0
6,5	1	0	1	1	2	3	3	0
6,5	1	0	0	1	1	3	3	0
6,7	1	1	0	1	2	2	2	0
6,5	1	0	0	1	1	2	3	1
7,4	1	0	0	0	0	3	3	0
6	0	1	0	0	1	1	1	0
NA	1	0	0	1	1	3	1	1
NA	0	0	0	1	1	1	2	0
6,4	1	0	0	1	1	2	3	0

NA	1	0	0	1	1	3	1	0
7	1	0	0	1	1	1	3	0
8,1	1	0	0	0	0	3	3	0
6,8	0	0	0	0	0	1	1	0
6,1	0	0	0	1	1	1	1	0
NA	0	0	0	0	0	1	1	0
7,9	0	0	0	1	1	1	1	1
NA	0	0	0	0	0	1	1	0
9,1	0	0	0	1	1	2	1	1
NA	0	0	0	1	1	1	2	1
NA	1	0	0	1	1	2	2	0
6,3	1	0	0	1	1	2	3	0
NA	1	0	0	1	1	3	3	0
8,2	1	1	1	1	3	3	3	0
7,5	1	0	0	1	1	2	3	0
9	1	0	0	1	1	3	3	0
5,4	1	0	0	1	1	3	3	0
NA	1	0	0	1	1	3	3	0
7,2	1	0	0	1	1	3	3	0
6,3	1	1	0	0	1	1	3	1
6,5	1	0	0	1	1	2	3	1
6,5	0	0	0	1	1	1	1	0
13	1	0	0	0	0	3	3	0
8,8	1	1	0	0	1	2	3	1
6	1	0	0	1	1	3	3	0
7,5	0	0	0	0	0	1	1	0
6,8	1	0	0	0	0	2	2	0
7,8	0	0	0	0	0	1	1	0
NA	0	0	0	0	0	1	1	1
NA	1	0	0	1	1	2	2	0
8,5	0	0	0	0	0	1	1	0
9	0	0	0	0	0	1	1	0
NA	1	0	0	1	1	3	3	0
7,2	1	0	0	1	1	3	2	0
6,7	1	0	0	1	1	3	3	0
NA	0	0	0	0	0	1	1	1
NA	1	0	0	1	1	3	2	1
6,1	0	0	0	0	0	1	1	1
NA	0	0	0	0	0	1	1	1
6,3	1	0	0	1	1	2	2	1
NA	0	0	0	0	0	1	1	0
NA	1	0	0	1	1	3	3	0
6,8	1	0	0	1	1	3	1	1
NA	0	0	0	0	0	1	1	1
NA	0	0	0	0	0	1	1	1
7,8	0	0	0	0	0	1	1	1
5,8	1	0	0	1	1	3	3	1
NA	0	0	0	0	0	1	1	0
NA	0	0	1	1	2	1	2	0
6,8	1	1	0	0	1	2	2	1

NA	0	0	0	0	0	1	1	0
9,8	1	1	0	0	1	2	3	0
NA	0	0	0	0	0	1	1	0
7,2	0	1	0	0	1	1	2	1
NA	0	0	0	1	1	1	2	0
NA	0	0	0	0	0	1	1	1
NA	0	0	0	0	0	1	1	0
6,7	0	1	0	1	2	1	2	1
6,8	0	0	1	1	2	1	2	0
NA	0	0	0	0	0	1	1	1
NA	0	0	0	0	0	1	1	0
NA	1	0	1	0	1	2	2	0
NA	0	0	0	0	0	1	1	0
6,5	0	0	0	1	1	1	1	0
6,9	1	0	0	1	1	2	3	0
7,7	0	0	0	1	1	2	1	1
9,4	1	0	0	0	0	1	3	1
11,3	0	0	0	0	0	1	1	1
NA	1	0	0	0	0	1	3	1
6,6	1	0	1	1	2	3	3	0
7	0	0	0	0	0	1	1	0
8,9	0	0	0	0	0	1	2	0
7,5	0	0	0	0	0	1	1	1
7,4	1	0	0	0	0	1	3	0
5,5	1	0	0	1	1	3	3	0
NA	1	0	0	0	0	1	3	0
7,3	1	0	0	1	1	3	3	0
7,7	1	0	0	1	1	3	2	0
6,1	1	0	0	0	0	3	2	0
7,9	1	0	0	1	1	3	3	0
6,6	1	0	0	1	1	2	2	0
7,4	1	1	0	1	2	3	2	0
6,4	0	0	0	0	0	1	1	0
6,4	0	0	0	0	0	1	2	0
6,2	0	0	0	1	1	1	1	0
NA	1	0	0	0	0	3	3	1
NA	0	0	0	0	0	1	1	1
7,4	1	0	0	1	1	3	3	0
7	0	0	0	1	1	1	1	0
6,9	0	0	0	0	0	1	1	0
6,5	1	0	0	0	0	3	3	0
6,3	0	0	0	0	0	1	1	1
6,9	1	0	0	1	1	3	3	0
NA	0	0	0	0	0	1	1	0
7,2	1	0	0	1	1	1	3	0
6,8	0	0	0	0	0	1	1	0
NA	0	0	0	0	0	1	1	0
5,9	1	0	0	1	1	3	2	1
5,7	1	0	0	1	1	2	3	1
7,3	1	0	0	1	1	3	2	0

7,2	0	0	0	0	0	1	1	0
8	1	0	0	1	1	3	3	0
7,6	0	0	0	0	0	1	1	0
6,7	0	0	0	1	1	1	1	1
15,1	1	1	0	0	1	3	1	1
NA	0	1	0	1	2	1	1	0
6,8	0	1	0	0	1	1	1	1
6,4	1	0	0	1	1	3	1	0
6,3	1	0	0	1	1	3	3	0
8,1	0	0	0	0	0	1	1	0
NA	0	0	0	0	0	1	1	0
NA	0	0	0	0	0	1	1	0
7,4	0	0	0	0	0	1	1	0
6	0	0	0	0	0	1	1	0
NA	0	0	0	0	0	1	1	0
NA	0	0	0	0	0	1	1	0
NA	0	0	0	0	0	1	1	1
NA	1	0	0	0	0	3	3	0
6,4	0	0	0	0	0	1	1	1
NA	0	0	0	0	0	1	1	0
NA	1	1	0	0	1	3	3	1
7,7	0	0	0	1	1	1	1	0
NA	0	0	0	0	0	1	1	0
NA	0	0	0	0	0	1	1	0
NA	0	0	0	0	0	1	1	0
6,2	1	0	0	0	0	3	3	1
NA	0	0	0	0	0	1	1	0
NA	1	0	0	0	0	3	3	1
NA	1	0	0	1	1	2	3	0
NA	0	0	0	0	0	1	1	0
7	0	0	0	0	0	1	1	1
7,6	1	0	0	1	1	3	2	1
NA	1	0	0	1	1	3	1	0
6,3	1	0	0	1	1	3	2	0
8,7	1	0	0	1	1	3	3	0
NA	1	0	0	0	0	3	3	0
7,6	1	0	0	1	1	1	3	0
6,9	1	0	0	0	0	3	3	0
7,3	1	0	0	1	1	3	3	1
7,6	0	0	0	0	0	1	1	0
8,2	0	0	0	1	1	1	1	1
6,8	1	1	1	0	2	2	3	0
6,8	1	0	0	1	1	3	3	0
8,4	0	0	0	1	1	2	1	0
7,2	1	1	0	0	1	1	3	1
7,8	0	0	0	0	0	1	1	1
13	0	0	0	0	0	1	1	1
6,8	0	1	0	1	2	1	2	1
NA	0	0	0	0	0	1	1	1
7	0	0	0	0	0	1	1	0

7,3	0	0	0	1	1	1	1	1
12,3	0	0	0	0	0	1	1	1
8,7	0	0	0	0	0	1	1	1
8,8	0	0	0	1	1	1	1	1
10,3	0	0	0	0	0	1	1	1
7,5	0	1	0	0	1	1	1	1
6,8	1	1	1	0	2	2	3	1
8	0	0	0	0	0	1	1	1
9	0	0	0	0	0	1	1	1
6,5	1	0	0	0	0	3	3	1
9,9	1	0	0	1	1	3	2	0
7,4	1	0	0	1	1	2	2	0
6,7	0	0	0	1	1	1	1	0
7,1	1	0	0	1	1	2	3	1
6,5	0	1	0	1	2	1	2	1
8,5	0	0	0	1	1	1	1	0
8,1	1	1	0	0	1	2	2	0
7,2	1	0	0	1	1	3	1	1
7,8	0	0	0	1	1	1	1	1
6,8	0	0	1	0	1	1	1	1
6,8	0	0	0	0	0	1	1	1
NA	1	0	0	1	1	2	2	1
NA	1	1	0	1	2	2	2	1
NA	0	0	0	1	1	2	1	0
7	0	0	0	0	0	1	1	0
6,3	1	0	0	1	1	2	2	1
NA	0	0	0	0	0	1	1	1
7	0	0	0	0	0	1	1	1
NA	0	0	0	1	1	1	2	1
NA	1	0	0	1	1	3	3	0
NA	0	0	0	0	0	1	1	0
NA	0	0	0	0	0	1	1	0
NA	0	0	0	0	0	1	1	1
NA	0	0	0	0	0	1	1	1
4,5	0	0	0	1	1	1	1	0
NA	1	0	0	1	1	2	2	0
NA	1	0	0	1	1	2	2	0
NA	0	0	0	1	1	1	1	1
7,8	0	0	0	0	0	1	1	0
6,2	1	1	0	0	1	2	3	1
NA	0	0	0	1	1	2	1	0
6,7	0	1	1	1	3	1	1	1
NA	0	0	0	0	0	1	1	1
NA	0	0	0	0	0	1	2	0
7,6	1	0	0	1	1	2	3	0
NA	0	0	0	0	0	1	1	0
NA	1	0	0	0	0	1	3	1
NA	0	0	0	0	0	1	1	0
7,6	0	0	0	1	1	1	1	0
7	1	0	0	1	1	3	3	1

6,9	0	0	0	1	1	1	1	1
NA	0	0	0	0	0	1	1	0
NA	1	0	0	1	1	2	2	1
NA	1	0	0	0	0	2	3	0
NA	0	0	0	1	1	1	1	0
NA	0	0	0	0	0	1	1	0
8,2	1	0	0	1	1	2	2	1
NA	1	1	0	0	1	2	2	1
NA	1	0	0	1	1	3	3	1
NA	0	0	0	0	0	1	1	0
NA	0	1	0	1	2	2	1	1
8	1	0	0	1	1	3	1	1
7,4	1	0	0	1	1	3	1	1
7	1	0	0	0	0	3	1	0
6,4	1	0	0	1	1	3	2	0
NA	1	0	0	1	1	3	2	0
NA	0	0	0	0	0	1	1	1
6,5	1	0	0	1	1	3	1	0
9,3	1	0	0	0	0	2	2	0
6,1	0	0	0	0	0	1	1	0
6,3	1	0	0	1	1	3	2	1
6,1	1	0	0	1	1	2	2	0
6,4	0	0	0	0	0	1	1	0
6,6	1	0	0	1	1	3	2	0
6,6	0	0	0	0	0	1	1	0
5,8	0	0	0	0	0	1	1	0
7,6	1	0	0	1	1	2	2	0
7,2	0	0	0	0	0	1	1	0
6,3	1	0	0	1	1	2	3	0
6,4	1	0	0	1	1	2	3	0
NA	1	0	0	1	1	3	2	0
NA	0	0	0	0	0	1	2	0
7,5	1	0	0	1	1	3	3	0
6,6	1	0	0	1	1	3	3	0
NA	1	0	0	1	1	3	3	0
NA	0	0	0	1	1	1	2	1
NA	1	0	0	1	1	3	3	1
NA	0	0	0	0	0	1	1	1
NA	1	0	0	1	1	3	3	0
7	1	0	0	1	1	3	3	0
6,4	1	0	0	1	1	2	2	0
7,5	1	0	0	0	0	3	1	1
6,9	0	0	0	0	0	1	2	1
8,7	1	1	0	0	1	1	3	1
6,6	1	0	0	1	1	2	3	0
11,8	1	1	0	0	1	3	3	0
7,6	0	0	0	1	1	1	1	0
9	0	0	0	0	0	1	1	0
NA	0	0	0	1	1	1	1	0
6,8	0	0	0	0	0	1	1	1

NA	1	0	0	1	1	1	3	0
NA	1	0	0	1	1	3	3	0
NA	0	0	0	0	0	1	1	0
6	1	1	0	0	1	1	3	0
7	1	0	0	1	1	2	2	0
7,2	0	0	0	1	1	1	2	0
6,4	0	0	0	0	0	1	2	0
7,4	0	0	0	0	0	1	2	0
7,4	1	1	0	0	1	3	2	1
6,8	1	0	0	0	0	2	2	1
6,3	0	0	0	0	0	1	2	0
6,1	1	1	0	1	2	3	3	1
8,3	0	0	0	0	0	1	1	0
7,8	1	0	0	1	1	3	3	1
8,3	1	0	0	1	1	2	2	1
7	0	0	0	0	0	1	1	0
8,2	0	0	0	0	0	1	1	1
6,8	0	0	0	1	1	1	1	1
7,5	0	0	0	0	0	1	1	1
6,3	1	0	0	1	1	2	2	1

ordr_regime	ordr_sport	priopat_ado	priopat_sport	priopat_stoptbc	bp_desequilibre
1	1	8	2	0	1
1	1	5	3	2	4
0	0	5	5	0	1
1	1	3	7	0	1
1	1	10	0	0	1
1	1	9	1	0	7
1	1	7	2	1	1
1	1	5	5	0	4
1	1	6	4	0	2
1	1	4	4	2	4
1	1	8	2	0	5
1	1	6	3	1	2
1	1	5	3	2	6
1	0	6	4	0	2
1	1	8	2	0	4
1	1	8	2	0	2
1	1	10	0	0	4
1	1	6	2	2	3
1	1	7	2	1	3
1	1	8	1	1	2
1	1	10	0	0	2
1	1	5	3	2	1
1	1	7	1	2	4
1	1	8	2	0	4
1	1	9	1	0	2
1	0	10	0	0	4
1	1	8	2	0	1
1	0	9	1	0	2
1	1	8	2	0	2
1	1	8	2	0	2
1	1	9	1	0	2
1	0	10	0	0	4
1	1	8	2	0	1
1	1	8	2	0	1
1	1	6	2	2	1
1	1	3	3	3	4
0	1	6	3	1	3
1	1	5	5	0	3
1	1	7	3	0	2
1	1	5	5	0	1
1	1	6	4	0	1
0	0	6	4	0	1
0	0	6	4	0	1
1	1	3	7	0	1
1	1	5	5	0	1
0	1	7	2	1	5
1	1	8	2	0	5
1	1	7	3	0	4
1	1	6	3	1	5

1	1	8	2	0	3
1	1	10	0	0	5
1	1	6	3	1	3
1	1	7	3	0	3
1	1	8	2	0	6
1	1	9	1	0	4
1	1	6	2	2	4
0	1	6	4	0	1
0	1	4	6	0	4
0	1	0	10	0	1
1	1	6	0	4	1
1	1	5	5	0	1
1	1	7	3	0	4
1	1	10	0	0	7
1	1	6	4	0	4
1	1	8	2	0	4
1	1	7	3	0	6
1	1	7	3	0	6
1	1	7	3	0	4
1	1	8	2	0	6
1	1	10	0	0	6
1	1	5	0	5	6
1	1	4	3	3	4
1	1	7	3	0	4
1	1	5	5	0	2
0	0	10	0	0	7
1	1	8	2	0	1
1	1	10	0	0	7
1	1	7	3	0	3
1	0	10	0	0	6
1	1	8	2	0	2
1	0	10	0	0	4
1	1	4	6	0	4
1	1	5	5	0	6
1	1	7	3	0	3
1	1	2	3	5	1
0	1	5	0	5	7
1	0	4	3	3	7
1	1	4	3	4	7
0	0	5	1	5	1
1	1	6	4	0	1
0	1	5	5	0	1
0	1	5	3	2	7
0	1	6	4	0	2
1	1	4	3	3	1
1	1	5	5	0	1
1	1	9	1	0	2
1	1	4	6	0	6
1	1	4	1	5	5
0	0	4	6	0	2

1	1	5	5	0	1
0	0	5	5	0	1
1	0	7	3	0	4
1	1	8	2	0	3
1	1	7	3	0	1
0	0	10	0	0	4
1	1	8	2	0	1
1	0	10	0	0	1
1	1	7	3	0	2
1	1	4	3	3	1
1	1	7	3	0	2
0	1	5	2	3	7
1	1	7	3	0	1
1	1	5	5	0	2
1	1	4	4	2	1
0	1	5	5	0	1
1	0	4	6	0	1
1	1	5	5	0	1
1	0	3	7	0	1
1	1	7	2	2	1
1	1	3	4	3	1
1	1	6	4	0	2
1	1	5	5	0	2
1	1	2	2	6	1
0	1	5	5	0	1
1	1	6	4	0	1
1	1	5	5	0	1
1	1	4	6	0	1
1	0	6	2	2	4
0	1	7	3	0	2
1	1	10	0	0	2
1	1	10	0	0	5
1	1	5	5	0	4
1	1	5	5	0	5
0	1	4	6	0	2
1	1	10	0	0	4
1	1	7	3	0	3
0	1	7	1	2	3
1	1	10	0	0	2
1	1	10	0	0	3
1	1	10	0	0	2
1	1	7	3	0	2
1	1	5	5	0	3
1	1	10	0	0	5
1	0	10	0	0	2
1	1	3	3	4	4
1	1	3	3	4	2
1	0	10	0	0	3
1	1	7	3	0	1
1	1	5	4	1	1

1	1	8	2	0	4
0	1	6	4	0	1
1	1	8	2	0	6
1	1	4	3	3	2
1	1	6	4	0	5
1	1	7	2	1	2
1	1	8	2	0	4
0	1	4	4	2	1
1	1	6	4	0	4
1	1	8	2	0	5
1	1	7	3	0	3
1	1	5	5	0	3
1	1	8	2	0	4
1	1	6	3	1	2
1	1	5	5	0	4
1	1	3	6	1	1
1	1	1	6	3	2
1	1	4	4	2	1
1	1	2	3	5	1
0	0	6	4	0	2
1	1	7	3	0	2
1	1	3	7	0	2
0	1	5	3	2	1
1	1	5	5	0	1
0	1	3	7	0	2
0	1	5	5	0	1
1	1	7	3	0	2
0	1	5	5	0	2
0	0	8	2	0	1
1	1	5	5	0	4
1	1	5	5	0	1
0	1	4	6	0	1
0	1	6	4	0	1
0	1	3	3	4	1
0	1	5	2	3	1
0	1	7	3	0	6
1	1	6	3	1	1
0	1	7	2	1	1
1	1	5	5	0	1
1	1	6	4	0	1
1	0	10	0	0	1
1	1	9	1	0	1
1	1	4	6	0	1
0	0	5	3	2	3
0	1	4	4	2	1
0	0	10	0	0	4
1	1	10	0	0	1
0	1	4	3	3	1
1	1	4	3	3	5
1	1	5	5	0	4

0	1	10	0	0	1
1	1	7	3	0	2
0	0	8	2	0	3
1	1	7	2	2	1
1	1	6	3	1	2
1	1	7	3	0	3
1	1	4	3	3	1
0	1	5	5	0	1
1	1	0	10	0	1
1	1	8	2	0	4
1	0	10	0	0	1
0	0	10	0	0	1
1	0	10	0	0	1
1	0	10	0	0	1
1	0	10	0	0	1
0	0	10	0	0	1
1	0	10	0	0	1
0	0	10	0	0	2
1	0	9	1	0	2
0	0	10	0	0	1
1	0	10	0	0	1
1	1	8	2	0	2
0	0	10	0	0	1
0	0	10	0	0	1
0	0	10	0	0	3
1	0	10	0	0	1
1	1	8	2	0	2
1	0	8	0	2	1
1	1	7	3	0	1
0	0	10	0	0	1
1	1	6	2	2	1
0	1	3	3	3	1
0	0	9	1	0	1
0	1	5	5	0	1
0	1	6	4	0	1
1	1	9	1	0	1
1	1	5	5	0	1
1	1	8	2	0	1
0	1	4	4	2	1
1	1	10	0	0	4
1	1	6	2	2	1
1	1	6	4	0	1
1	1	6	4	0	1
1	1	8	2	0	1
1	1	4	4	2	1
1	1	6	3	1	5
1	1	7	1	2	7
1	1	7	2	1	5
1	0	9	0	1	6
1	0	9	1	0	6

1	1	6	2	2	4
1	1	4	2	4	4
1	1	7	0	3	3
1	1	6	1	3	2
1	1	6	1	3	7
1	1	4	3	3	5
1	1	6	4	0	5
1	1	8	2	0	6
1	1	8	2	0	4
1	0	5	3	2	5
1	1	5	3	2	1
0	1	5	5	0	7
1	0	5	3	2	1
1	1	5	3	2	1
0	1	3	3	4	4
1	1	9	1	0	4
0	1	6	4	0	3
1	1	5	3	2	1
1	0	4	1	5	6
1	1	4	4	2	3
1	1	4	1	5	2
1	1	2	6	2	5
1	1	3	4	3	4
1	1	5	5	0	3
1	1	7	3	0	4
1	1	3	4	3	1
1	1	6	1	3	4
1	1	10	0	0	4
1	1	6	2	2	4
1	1	6	4	0	3
1	1	10	0	0	5
1	1	8	2	0	4
1	1	9	1	0	3
1	0	8	1	1	4
1	1	8	2	0	3
1	1	9	1	0	2
1	1	3	7	0	2
1	1	7	3	0	2
1	1	7	3	0	2
0	1	6	4	0	2
1	1	7	3	0	2
1	1	2	7	1	1
1	1	8	1	1	4
1	1	9	1	0	2
1	1	6	4	0	2
1	1	10	0	0	4
1	1	9	1	0	1
1	1	10	0	0	5
1	1	9	1	0	4
1	1	4	4	2	1

1	1	4	2	4	1
1	1	8	2	0	1
0	1	0	8	2	1
1	0	8	2	0	4
1	1	8	2	0	3
1	1	4	3	3	1
0	1	6	3	1	1
0	1	4	2	4	1
0	1	4	2	4	1
1	1	8	2	0	1
0	1	2	8	0	1
0	1	5	5	0	1
1	1	3	3	4	1
1	1	6	4	0	1
1	1	6	4	0	1
0	1	0	10	0	1
0	1	4	2	4	1
1	1	4	3	4	3
0	1	8	2	0	2
1	1	1	5	4	1
0	1	3	5	2	1
1	1	5	5	0	1
1	1	4	6	0	1
1	1	5	5	0	1
0	0	10	0	0	1
1	0	10	0	0	1
0	1	3	6	1	6
1	1	6	4	0	2
1	1	8	2	0	2
0	1	5	5	0	1
1	0	8	2	0	1
0	1	5	5	0	1
0	1	5	5	0	1
1	0	7	3	0	1
1	0	5	5	0	1
1	1	6	2	2	2
1	1	6	4	0	1
1	1	3	4	3	1
1	0	10	0	0	1
1	0	7	3	0	1
1	1	5	5	0	1
1	1	3	5	2	7
1	1	5	2	3	4
1	1	4	2	4	7
1	1	5	5	0	3
0	0	2	8	0	1
1	1	4	6	0	1
1	1	8	2	0	2
1	1	3	7	0	2
1	1	7	3	0	1

1	1	5	5	0	1
1	1	5	5	0	1
1	1	8	2	0	4
1	1	5	5	0	1
0	0	8	2	0	1
0	1	8	2	0	1
1	1	7	3	0	1
0	1	8	2	0	1
1	1	5	5	0	1
1	1	6	2	2	1
0	1	9	1	0	4
0	1	4	3	3	1
1	1	5	4	1	4
1	1	4	4	3	1
1	1	5	4	1	2
1	1	6	4	0	3
1	1	7	3	0	2
1	1	5	4	1	3
1	1	6	4	0	5
1	1	6	4	0	3

bp_hypoglycemie	bp_fatigue	bp_blessure	bp_cardiovasc	bp_meforme	bp_diabete
1	7	1	4	7	1
3	2	4	3	1	1
1	1	1	1	1	1
4	2	2	4	4	2
4	7	7	7	7	7
7	7	7	7	7	7
1	1	1	1	1	1
6	6	1	4	5	3
2	3	1	5	4	1
6	6	2	5	4	1
6	6	2	6	6	4
5	5	2	5	4	2
7	6	2	6	4	2
2	2	4	4	6	6
5	6	7	6	6	4
4	4	5	5	6	2
5	7	7	5	7	4
3	7	7	4	7	1
3	3	6	5	6	4
2	3	5	4	7	1
5	7	7	4	7	2
2	3	6	4	4	1
5	5	5	6	6	1
6	6	6	6	6	2
4	5	3	5	5	1
5	5	6	6	6	2
1	1	1	4	5	1
3	7	7	4	6	1
3	2	3	3	5	1
2	2	6	6	6	1
4	4	5	5	2	1
1	5	5	6	7	1
2	6	6	6	6	1
3	2	2	2	6	1
2	2	6	4	7	2
5	7	4	7	7	4
4	5	2	3	2	4
5	4	3	4	5	3
4	4	5	5	6	5
3	3	1	2	3	1
3	2	1	3	2	1
2	2	1	1	1	1
1	1	1	1	1	1
3	3	2	3	2	3
1	1	1	1	2	1
3	6	4	4	3	5
6	6	3	4	3	4
4	4	3	5	4	4
4	4	5	4	3	4

5	6	2	2	5	4
5	7	6	5	4	4
3	2	2	4	3	3
3	3	2	2	2	3
6	5	5	6	5	6
5	3	4	5	4	7
4	1	1	1	7	1
1	1	1	1	1	1
5	6	6	6	5	6
3	1	1	1	1	1
1	3	3	1	1	1
1	1	1	1	4	1
7	6	2	6	4	4
7	5	2	7	4	7
6	6	2	4	4	4
7	6	1	1	1	4
6	6	2	2	4	4
7	5	1	1	3	4
5	1	1	1	3	4
6	2	2	7	4	4
4	6	4	3	5	7
6	1	1	1	1	4
7	7	2	5	6	2
4	6	1	4	3	4
2	2	2	2	2	2
7	5	4	7	4	4
4	5	1	1	6	1
7	6	4	4	6	7
7	4	4	4	5	1
5	6	2	7	4	4
2	2	2	4	4	2
4	4	4	4	4	7
4	4	4	4	4	1
4	5	5	6	5	6
3	4	4	4	3	3
1	1	1	1	2	1
4	1	4	1	4	1
7	7	7	7	7	7
7	7	7	7	7	7
1	5	1	7	7	4
7	7	4	7	7	7
1	1	1	1	1	1
7	1	1	1	1	1
4	3	1	2	2	1
1	1	1	1	1	1
1	1	1	1	1	1
1	1	1	1	1	1
7	5	2	1	4	4
1	2	1	5	5	5
2	2	2	1	1	1

4	4	7	4	4	1
4	1	1	1	1	1
5	5	4	5	4	3
4	5	2	2	6	3
5	4	1	1	3	1
4	7	6	2	4	4
1	4	1	4	5	1
1	1	1	1	5	1
2	5	6	3	6	1
3	3	3	5	5	1
1	2	1	2	3	1
2	2	2	2	4	4
1	1	1	1	5	1
4	1	1	3	1	1
1	1	1	1	1	1
1	1	1	1	1	1
1	1	1	1	1	1
1	3	1	1	2	1
1	1	1	1	2	1
1	1	7	3	1	3
1	1	1	1	1	1
1	1	2	1	2	1
3	3	3	1	4	3
1	1	1	1	3	1
1	1	1	1	1	1
1	1	1	2	6	2
2	4	2	4	6	2
2	6	2	4	6	2
5	6	6	3	7	4
2	5	3	3	5	4
3	5	4	2	6	6
5	5	4	6	5	5
4	4	4	6	4	3
6	6	5	4	5	4
2	4	3	2	6	2
4	5	3	4	6	2
3	3	4	4	2	2
3	5	4	5	3	3
2	5	4	2	5	2
3	4	5	4	5	6
2	4	3	4	6	5
2	2	2	1	3	2
3	5	4	3	6	3
3	3	4	5	5	6
2	5	4	6	2	3
3	6	6	6	6	4
2	4	2	4	3	2
2	2	3	4	2	3
4	4	1	1	2	1
2	2	1	1	1	1

4	5	4	5	4	4
3	1	1	3	1	1
6	5	2	2	6	4
2	2	2	2	2	2
5	5	2	2	4	5
2	6	5	2	7	3
4	6	6	4	6	4
2	1	1	1	1	1
4	3	2	2	2	4
6	6	6	5	7	6
3	6	2	4	6	3
3	5	2	5	5	3
5	6	5	2	6	4
3	5	2	2	3	1
4	6	4	6	6	3
1	2	1	1	2	5
2	2	6	2	3	6
1	4	2	6	4	5
1	1	1	1	5	1
2	1	5	1	2	1
4	6	5	3	7	4
5	6	3	5	6	2
1	1	1	1	2	1
5	5	2	4	6	1
2	5	5	2	5	5
4	1	1	1	4	1
6	7	1	1	2	6
3	7	1	1	2	5
1	6	7	1	1	1
1	1	1	1	3	1
4	6	1	3	7	1
2	3	1	4	5	1
4	3	5	1	3	1
1	1	1	1	1	1
3	3	3	3	3	3
7	7	6	7	4	4
1	1	1	1	6	1
6	1	1	1	1	1
1	1	1	1	1	1
1	1	2	2	3	1
1	1	1	1	1	1
1	1	1	1	5	1
1	1	1	1	1	1
2	5	1	1	4	1
1	1	1	1	1	1
7	4	7	4	7	4
1	3	1	1	1	1
4	6	6	3	3	1
4	3	3	2	3	2
2	2	4	2	4	4

1	6	1	2	1	1
2	2	2	2	2	2
2	5	4	3	5	2
4	2	5	1	1	1
2	2	2	2	4	4
4	3	1	1	4	1
2	3	1	4	3	1
1	1	1	1	1	1
1	1	1	1	1	1
5	5	5	6	6	5
1	5	1	1	2	1
1	1	1	1	6	1
1	4	1	1	4	1
1	1	1	1	6	1
1	6	1	1	6	1
1	2	1	1	1	1
1	2	1	1	3	1
3	3	1	1	1	5
2	2	1	1	3	1
1	6	1	1	6	1
1	1	1	1	1	1
2	2	2	1	2	1
1	1	1	1	5	1
3	4	1	1	4	4
3	4	1	1	4	3
1	1	1	1	1	1
2	1	1	1	2	2
1	1	1	1	1	1
1	1	1	1	1	2
1	4	1	1	3	1
1	1	1	6	6	1
3	1	6	6	3	1
1	6	6	1	1	2
1	1	1	1	1	1
1	1	1	1	4	5
1	1	1	1	1	1
5	4	1	1	1	1
4	2	1	1	1	1
5	4	1	1	1	1
4	6	6	1	5	1
1	1	1	1	1	1
4	3	3	1	1	1
1	3	2	2	1	1
1	4	3	1	1	1
3	3	3	4	1	1
7	7	3	6	6	6
7	7	3	7	7	4
5	5	3	5	5	5
6	6	4	7	7	5
6	6	5	7	7	5

7	5	1	3	3	1
5	7	1	7	7	3
6	7	6	6	7	4
6	4	2	2	6	2
7	7	7	7	7	4
5	5	3	3	6	3
5	4	2	2	2	2
6	6	6	6	4	5
4	4	3	3	3	3
3	3	3	3	3	3
5	2	2	2	2	6
7	7	7	7	7	7
1	1	1	1	1	1
2	2	1	4	3	4
3	3	4	3	4	4
4	5	4	4	5	5
3	4	4	5	5	5
3	3	7	2	1	6
7	6	1	6	6	6
5	5	4	4	4	3
2	5	4	3	6	2
3	5	4	4	6	2
6	6	6	6	5	2
5	5	4	4	7	3
4	6	6	6	6	3
1	4	3	4	5	2
6	6	5	6	7	3
5	6	6	6	6	3
5	4	5	5	6	3
5	6	5	6	6	3
6	7	7	7	7	4
4	2	2	3	5	2
4	6	6	6	7	3
3	7	7	6	7	4
3	4	3	5	6	3
5	5	3	5	5	2
2	4	2	5	5	2
2	3	2	2	5	2
3	4	2	4	5	2
2	1	2	3	2	2
2	4	2	3	4	2
1	2	1	1	4	1
4	5	5	5	6	2
3	5	5	6	6	2
5	1	1	2	1	2
4	6	6	6	6	4
2	1	1	3	3	1
5	6	6	6	6	6
5	3	2	2	4	3
1	1	1	1	1	1

1	1	1	1	7	1
1	2	1	1	1	1
1	3	2	2	3	1
4	5	3	4	6	4
1	4	1	1	3	3
4	5	1	3	6	1
3	3	1	1	3	1
3	3	1	5	2	6
1	1	1	1	1	1
1	1	1	1	1	1
1	1	1	1	1	1
1	1	1	4	4	1
1	4	1	1	4	1
5	2	1	1	1	1
1	1	1	1	1	1
1	1	1	1	1	1
1	6	6	1	5	2
3	3	3	3	3	3
2	3	2	2	3	2
1	1	1	1	1	1
1	1	1	1	1	1
1	7	7	7	7	1
1	1	1	4	6	1
1	4	1	1	1	1
2	4	1	2	5	1
1	1	1	1	1	1
1	2	2	1	3	2
2	1	1	2	2	2
7	5	1	5	5	2
1	1	1	1	1	1
1	4	1	1	1	1
1	1	1	1	4	1
1	1	2	1	5	1
1	3	1	3	1	1
5	6	5	1	7	1
2	2	5	6	5	2
1	2	1	1	4	1
1	1	1	1	2	1
1	1	1	1	4	1
1	5	1	6	6	1
1	2	1	1	6	1
4	6	2	5	7	4
4	4	4	4	4	4
6	5	7	7	5	6
4	6	1	4	4	5
7	1	1	1	1	1
1	4	3	5	4	1
1	1	2	1	2	1
6	6	3	2	5	1
6	5	4	4	4	3

4	4	1	1	2	1
3	6	6	5	7	1
2	2	1	4	2	6
3	3	1	1	3	1
2	1	1	1	3	2
4	4	1	1	6	4
1	1	1	1	3	1
4	1	2	1	1	1
1	1	2	7	4	1
1	1	4	1	3	2
1	6	1	5	1	5
4	2	5	6	4	1
5	7	2	3	7	5
2	1	3	3	2	1
5	5	4	4	3	4
5	5	4	4	3	4
5	6	5	4	7	5
5	6	4	4	7	5
6	6	4	5	7	4
2	4	2	1	3	2

bp_hyperglycemie	bp_sante	bp_climat	bp_horaire	bapad_pat	bp	hba1c_grp	hba1c_grp_01
1	1	7	1	32	2,91	1	1
3	1	2	1	25	2,27	1	1
1	1	4	3	16	1,45	0	0
2	3	6	7	37	3,36	1	1
4	7	7	1	59	5,36	1	1
1	7	7	1	65	5,91	2	0
1	1	5	1	15	1,36	0	0
2	5	5	1	42	3,82	0	0
1	1	7	4	31	2,82	0	0
1	4	7	1	41	3,73	0	0
2	4	7	1	49	4,45	0	0
2	2	7	1	37	3,36	1	1
1	5	7	1	47	4,27	1	1
1	2	1	1	31	2,82	0	0
4	7	6	4	59	5,36	2	0
1	5	5	7	46	4,18	0	0
4	7	7	1	58	5,27	2	0
1	7	7	1	48	4,36	1	1
1	7	1	2	41	3,73	0	0
1	5	2	7	39	3,55	1	1
2	7	4	1	48	4,36	0	0
1	2	5	1	30	2,73	0	0
1	4	5	1	43	3,91	0	0
2	7	5	1	51	4,64	0	0
1	7	1	1	35	3,18	2	0
2	7	5	1	49	4,45	0	0
1	2	3	1	21	1,91	0	0
1	7	5	1	44	4	1	1
1	2	2	7	31	2,82	1	1
1	3	1	1	31	2,82	0	0
1	7	1	1	33	3	0	0
1	7	1	1	39	3,55	2	0
1	7	5	1	42	3,82	1	1
1	2	1	7	28	2,55	0	0
2	7	1	6	40	3,64	2	0
5	6	4	3	56	5,09	1	1
1	5	5	5	39	3,55	2	0
2	2	6	1	38	3,45	1	1
3	6	6	5	51	4,64	1	1
2	6	6	4	32	2,91	2	0
1	6	6	5	31	2,82	1	1
1	7	1	1	19	1,73	0	0
1	7	1	1	17	1,55	0	0
1	1	1	1	21	1,91	2	0
1	1	3	1	14	1,27	1	1
2	4	5	5	46	4,18	1	1
3	5	5	2	46	4,18	1	1
2	5	6	3	44	4	2	0
6	2	5	5	47	4,27	1	1

2	2	5	5	41	3,73	2	0
4	5	5	2	52	4,73	2	0
1	2	4	4	31	2,82	1	1
1	2	4	5	30	2,73	0	0
6	6	7	4	62	5,64	1	1
4	4	6	4	50	4,55	2	0
4	1	7	4	35	3,18	1	1
1	1	3	1	13	1,18	0	0
6	1	2	1	48	4,36	2	0
1	1	3	1	15	1,36	2	0
1	4	6	1	23	2,09	2	0
1	1	6	1	19	1,73	0	0
4	1	4	4	46	4,18	1	1
7	7	5	4	62	5,64	2	0
4	4	6	3	47	4,27	1	1
7	4	6	2	43	3,91	2	0
6	4	6	5	51	4,64	2	0
1	1	1	7	37	3,36	0	0
1	1	5	7	33	3	0	0
2	7	7	7	54	4,91	1	1
4	6	6	7	58	5,27	2	0
4	1	1	7	33	3	2	0
1	4	5	1	44	4	0	0
4	1	6	7	44	4	1	1
4	7	7	4	36	3,27	2	0
4	7	7	4	60	5,45	2	0
5	1	7	7	39	3,55	2	0
4	7	6	4	62	5,64	0	0
4	2	6	7	47	4,27	0	0
5	7	7	3	56	5,09	2	0
2	6	4	4	34	3,09	2	0
4	4	6	7	52	4,73	2	0
4	1	3	1	34	3,09	0	0
7	6	5	4	59	5,36	1	1
3	3	3	3	36	3,27	0	0
1	2	3	4	18	1,64	0	0
1	4	4	1	32	2,91	1	1
7	7	7	7	77	7	1	1
7	4	7	7	74	6,73	2	0
7	4	1	1	39	3,55	2	0
7	7	4	1	59	5,36	0	0
1	1	1	1	11	1	0	0
7	1	5	1	33	3	0	0
1	1	4	1	22	2	0	0
1	1	1	1	11	1	0	0
1	1	1	1	11	1	1	1
1	1	1	1	12	1,09	0	0
1	5	7	1	43	3,91	2	0
1	1	2	1	29	2,64	2	0
1	1	1	1	15	1,36	0	0

1	4	1	1	32	2,91	2	0
3	1	1	1	16	1,45	0	0
4	4	1	1	40	3,64	1	1
1	1	1	5	33	3	0	0
1	1	5	1	24	2,18	0	0
4	7	1	1	44	4	2	0
1	5	1	1	25	2,27	1	1
1	5	1	1	19	1,73	2	0
1	4	1	1	32	2,91	1	1
3	2	2	1	29	2,64	2	0
1	2	4	1	20	1,82	2	0
3	4	3	2	35	3,18	0	0
1	6	3	1	22	2	2	0
1	1	4	1	20	1,82	1	1
1	1	1	1	11	1	1	1
1	1	1	1	11	1	1	1
1	1	1	1	11	1	0	0
1	1	6	1	19	1,73	2	0
1	1	2	1	13	1,18	1	1
1	1	1	1	21	1,91	0	0
1	1	5	1	15	1,36	0	0
1	2	2	1	16	1,45	0	0
2	2	3	4	30	2,73	1	1
1	2	2	7	21	1,91	1	1
1	3	1	1	13	1,18	0	0
1	2	3	1	21	1,91	1	1
2	2	2	6	33	3	0	0
2	4	3	7	39	3,55	1	1
4	7	6	4	56	5,09	2	0
4	3	7	4	42	3,82	2	0
2	6	6	1	43	3,91	1	1
3	6	6	1	51	4,64	1	1
4	5	6	7	51	4,64	2	0
3	4	6	4	52	4,73	1	1
2	4	5	7	39	3,55	0	0
3	2	5	5	43	3,91	2	0
2	2	2	3	30	2,73	2	0
4	3	5	5	43	3,91	0	0
2	4	6	2	36	3,27	2	0
5	5	5	3	48	4,36	0	0
6	5	6	2	45	4,09	2	0
2	2	4	6	28	2,55	2	0
3	5	5	6	46	4,18	0	0
4	4	5	2	46	4,18	2	0
2	2	4	2	34	3,09	2	0
5	7	6	7	60	5,45	1	1
2	5	5	2	33	3	0	0
4	5	6	7	41	3,73	2	0
1	1	1	5	22	2	2	0
1	1	2	2	15	1,36	0	0

4	5	5	1	45	4,09	2	0
1	1	4	1	18	1,64	1	1
2	2	6	6	47	4,27	2	0
2	2	4	4	26	2,36	1	1
5	2	3	3	41	3,73	2	0
2	6	6	6	47	4,27	2	0
4	6	5	1	50	4,55	2	0
1	1	4	4	18	1,64	0	0
4	1	3	1	30	2,73	0	0
6	7	5	5	64	5,82	2	0
4	5	5	4	45	4,09	2	0
3	6	2	1	38	3,45	2	0
4	6	4	1	47	4,27	2	0
1	1	1	4	25	2,27	0	0
3	7	7	1	51	4,64	0	0
1	2	4	1	21	1,91	1	1
2	7	6	1	39	3,55	1	1
3	4	2	2	34	3,09	1	1
1	2	2	7	23	2,09	2	0
2	2	5	1	24	2,18	0	0
5	7	6	1	50	4,55	0	0
2	6	1	1	39	3,55	1	1
1	1	5	7	22	2	1	1
1	7	6	1	39	3,55	1	1
5	7	6	1	45	4,09	0	0
1	1	2	1	18	1,64	2	0
1	1	1	3	31	2,82	1	1
1	2	6	1	31	2,82	1	1
1	7	2	1	29	2,64	0	0
1	2	2	1	18	1,64	1	1
1	4	7	1	36	3,27	0	0
1	1	2	1	22	2	1	1
1	2	5	5	31	2,82	0	0
1	1	4	7	20	1,82	0	0
3	3	3	3	31	2,82	0	0
3	5	4	7	60	5,45	2	0
1	1	1	2	17	1,55	2	0
1	1	1	1	16	1,45	1	1
1	1	6	6	21	1,91	0	0
1	2	3	7	24	2,18	0	0
1	1	4	7	20	1,82	0	0
1	1	1	1	15	1,36	0	0
1	1	4	1	14	1,27	0	0
1	2	3	1	24	2,18	2	0
1	1	1	1	11	1	1	1
1	7	4	7	56	5,09	0	0
1	3	1	1	15	1,36	2	0
1	4	5	1	35	3,18	0	0
4	5	6	2	39	3,55	0	0
1	5	4	2	34	3,09	1	1

1	2	6	1	23	2,09	1	1
2	4	2	2	24	2,18	1	1
2	5	3	1	35	3,18	1	1
4	2	2	1	24	2,18	0	0
3	7	1	1	30	2,73	1	1
1	1	7	4	30	2,73	2	0
1	2	5	1	24	2,18	0	0
1	1	7	1	17	1,55	0	0
1	1	1	1	11	1	0	0
5	5	4	1	51	4,64	1	1
1	7	2	1	23	2,09	2	0
1	7	1	1	22	2	2	0
1	7	3	1	25	2,27	1	1
1	7	1	1	22	2	0	0
1	7	1	1	27	2,45	2	0
1	2	1	1	13	1,18	2	0
1	7	2	1	21	1,91	2	0
1	1	1	1	20	1,82	2	0
1	5	3	1	22	2	0	0
1	7	1	1	27	2,45	2	0
1	4	4	1	17	1,55	2	0
1	2	1	3	19	1,73	1	1
1	7	3	1	23	2,09	2	0
1	7	3	1	30	2,73	2	0
1	5	4	1	30	2,73	2	0
1	1	3	1	13	1,18	0	0
1	1	2	6	21	1,91	2	0
1	1	1	1	11	1	2	0
1	1	2	1	13	1,18	2	0
1	7	1	1	22	2	2	0
1	4	1	1	24	2,18	0	0
1	1	6	1	30	2,73	1	1
1	5	1	1	26	2,36	2	0
1	1	1	1	11	1	0	0
1	7	5	1	28	2,55	1	1
1	1	1	1	11	1	2	0
1	4	1	7	27	2,45	1	1
1	1	1	1	15	1,36	0	0
1	1	3	1	20	1,82	1	1
1	4	4	1	37	3,36	1	1
1	1	1	7	17	1,55	1	1
1	1	3	6	25	2,27	0	0
1	1	4	4	21	1,91	0	0
1	1	5	1	20	1,82	1	1
1	1	4	3	25	2,27	1	1
3	3	1	1	48	4,36	1	1
4	4	1	4	55	5	1	1
1	1	4	4	43	3,91	0	0
4	5	5	5	60	5,45	2	0
3	5	5	1	56	5,09	0	0

1	2	2	1	30	2,73	1	1
1	1	1	4	41	3,73	1	1
1	5	2	7	54	4,91	1	1
1	1	1	1	28	2,55	1	1
1	7	1	3	58	5,27	1	1
1	1	2	3	37	3,36	1	1
2	2	2	2	30	2,73	0	0
3	5	2	1	50	4,55	1	1
2	2	2	1	31	2,82	1	1
3	3	3	3	35	3,18	0	0
2	6	2	1	31	2,82	1	1
7	7	7	7	77	7	1	1
1	1	6	1	16	1,45	0	0
2	1	6	1	27	2,45	1	1
5	4	5	1	40	3,64	0	0
4	4	5	2	46	4,18	1	1
4	3	3	3	42	3,82	1	1
1	1	1	1	27	2,45	1	1
6	1	3	1	49	4,45	1	1
2	6	7	6	49	4,45	0	0
1	2	7	7	41	3,73	0	0
2	4	6	4	45	4,09	2	0
2	6	7	3	53	4,82	2	0
2	5	6	5	49	4,45	2	0
3	5	6	3	52	4,73	0	0
1	3	6	5	35	3,18	0	0
3	5	6	6	57	5,18	2	0
3	5	6	6	56	5,09	0	0
3	4	6	6	51	4,64	2	0
2	3	5	4	48	4,36	2	0
2	7	7	2	61	5,55	2	0
2	6	6	1	37	3,36	2	0
3	6	7	1	52	4,73	2	0
4	7	7	1	57	5,18	2	0
3	4	5	1	40	3,64	0	0
2	5	6	1	41	3,73	2	0
2	2	5	1	32	2,91	2	0
2	3	5	7	35	3,18	2	0
2	2	5	2	33	3	1	1
2	2	6	1	25	2,27	0	0
2	5	6	1	33	3	2	0
1	1	5	6	24	2,18	0	0
2	6	6	1	46	4,18	2	0
2	4	6	1	42	3,82	2	0
1	2	5	5	27	2,45	1	1
4	6	7	1	54	4,91	2	0
1	2	4	6	25	2,27	2	0
4	6	6	1	57	5,18	2	0
2	4	4	1	34	3,09	1	1
1	1	1	1	11	1	0	0

1	6	5	1	26	2,36	0	0
1	1	1	1	12	1,09	2	0
1	3	6	5	28	2,55	2	0
4	4	3	7	48	4,36	2	0
2	6	7	1	32	2,91	2	0
3	3	2	1	30	2,73	2	0
1	4	3	3	24	2,18	1	1
1	3	4	1	30	2,73	2	0
1	1	1	1	11	1	2	0
1	4	1	4	17	1,55	2	0
1	1	1	1	11	1	2	0
4	3	1	1	22	2	1	1
1	1	1	1	17	1,55	1	1
1	5	5	7	30	2,73	0	0
1	3	6	1	18	1,64	0	0
1	1	4	1	14	1,27	2	0
1	5	5	1	34	3,09	2	0
3	3	3	3	33	3	0	0
1	2	2	1	22	2	1	1
1	1	1	1	11	1	0	0
1	1	1	1	11	1	0	0
1	1	7	1	41	3,73	0	0
1	1	2	1	20	1,82	0	0
1	1	1	1	14	1,27	0	0
2	2	1	2	23	2,09	0	0
1	7	1	1	17	1,55	0	0
1	7	1	1	27	2,45	1	1
2	2	7	1	24	2,18	1	1
1	1	6	1	36	3,27	0	0
1	1	1	1	11	1	0	0
1	7	5	1	24	2,18	2	0
1	7	7	1	26	2,36	2	0
1	1	1	1	16	1,45	1	1
1	5	5	1	23	2,09	0	0
1	7	4	1	39	3,55	2	0
2	2	6	1	35	3,18	2	0
1	6	6	3	27	2,45	2	0
1	5	5	7	26	2,36	2	0
1	4	3	1	19	1,73	2	0
1	6	4	1	33	3	0	0
1	4	6	1	25	2,27	0	0
5	7	3	1	51	4,64	1	1
4	4	4	4	44	4	0	0
6	6	5	3	63	5,73	1	1
2	4	5	2	40	3,64	0	0
1	1	1	1	17	1,55	1	1
1	4	4	1	29	2,64	1	1
1	1	2	1	15	1,36	1	1
2	6	3	1	37	3,36	2	0
1	4	3	1	36	3,27	0	0

1	1	1	6	23	2,09	2	0
1	4	4	1	39	3,55	2	0
6	7	1	1	36	3,27	2	0
1	1	3	5	23	2,09	0	0
1	1	3	1	17	1,55	0	0
1	4	7	1	34	3,09	1	1
1	1	1	1	13	1,18	0	0
1	4	3	6	25	2,27	1	1
1	1	7	1	27	2,45	1	1
3	1	4	1	22	2	0	0
2	1	7	1	34	3,09	0	0
1	1	7	1	33	3	0	0
2	6	3	1	45	4,09	1	1
1	2	1	1	18	1,64	1	1
1	2	2	2	34	3,09	1	1
1	6	5	3	43	3,91	0	0
2	2	4	3	45	4,09	1	1
2	4	3	7	50	4,55	0	0
2	2	6	7	54	4,91	1	1
1	2	3	3	26	2,36	0	0

hba1c_grp_02	ado_priopat	sport_priopat	stoptbc_priopat	priopat
0	1	0	0	100
0	1	0	0	100
0	1	1	0	110
0	0	1	0	10
0	1	0	0	100
1	1	0	0	100
0	1	0	0	100
0	1	1	0	110
0	1	0	0	100
0	1	1	0	110
0	1	0	0	100
0	1	0	0	100
0	1	0	0	100
0	1	0	0	100
1	1	0	0	100
0	1	0	0	100
1	1	0	0	100
0	1	0	0	100
0	1	0	0	100
0	1	0	0	100
0	1	0	0	100
0	1	0	0	100
0	1	0	0	100
0	1	0	0	100
1	1	0	0	100
0	1	0	0	100
0	1	0	0	100
0	1	0	0	100
0	1	0	0	100
0	1	0	0	100
0	1	0	0	100
1	1	0	0	100
0	1	0	0	100
0	1	0	0	100
1	1	0	0	100
0	0	0	1	1
1	1	0	0	100
0	1	1	0	110
0	1	0	0	100
1	1	1	0	110
0	1	0	0	100
0	1	0	0	100
0	1	0	0	100
1	0	1	0	10
0	1	1	0	110
0	1	0	0	100
0	1	0	0	100
1	1	0	0	100
0	1	0	0	100

1	1	0	0	100
1	1	0	0	100
0	1	0	0	100
0	1	0	0	100
0	1	0	0	100
1	1	0	0	100
0	1	0	0	100
0	1	0	0	100
1	0	1	0	10
1	0	1	0	10
1	1	0	0	100
0	1	1	0	110
0	1	0	0	100
1	1	0	0	100
0	1	0	0	100
1	1	0	0	100
1	1	0	0	100
0	1	0	0	100
0	1	0	0	100
0	1	0	0	100
1	1	0	0	100
1	1	0	1	101
0	1	0	0	100
0	1	0	0	100
1	1	1	0	110
1	1	0	0	100
1	1	0	0	100
0	1	0	0	100
0	1	0	0	100
1	1	0	0	100
1	1	0	0	100
1	1	0	0	100
0	0	1	0	10
0	1	1	0	110
0	1	0	0	100
0	0	0	1	1
0	1	0	1	101
0	1	0	0	100
1	1	0	1	101
1	1	0	0	100
0	1	0	0	100
0	1	1	0	110
0	1	0	0	100
0	1	0	0	100
0	1	0	0	100
0	1	1	0	110
0	1	0	0	100
1	0	1	0	10
1	0	0	1	1
0	0	1	0	10

1	1	1	0	110
0	1	1	0	110
0	1	0	0	100
0	1	0	0	100
0	1	0	0	100
1	1	0	0	100
0	1	0	0	100
1	1	0	0	100
0	1	0	0	100
1	1	0	0	100
1	1	0	0	100
0	1	0	0	100
1	1	0	0	100
0	1	1	0	110
0	1	1	0	110
0	1	1	0	110
0	0	1	0	10
1	1	1	0	110
0	0	1	0	10
0	1	0	0	100
0	0	1	0	10
0	1	0	0	100
0	1	1	0	110
0	0	0	1	1
0	1	1	0	110
0	1	0	0	100
0	1	1	0	110
0	0	1	0	10
1	1	0	0	100
1	1	0	0	100
0	1	0	0	100
0	1	0	0	100
1	1	1	0	110
0	1	1	0	110
0	0	1	0	10
1	1	0	0	100
1	1	0	0	100
0	1	0	0	100
1	1	0	0	100
0	1	0	0	100
1	1	0	0	100
1	1	0	0	100
0	1	1	0	110
1	1	0	0	100
1	1	0	0	100
0	0	0	1	1
0	0	0	1	1
1	1	0	0	100
1	1	0	0	100
0	1	0	0	100

1	1	0	0	100
0	1	0	0	100
1	1	0	0	100
0	1	0	0	100
1	1	0	0	100
1	1	0	0	100
1	1	0	0	100
0	1	1	0	110
0	1	0	0	100
1	1	0	0	100
1	1	0	0	100
1	1	1	0	110
1	1	0	0	100
0	1	0	0	100
0	1	1	0	110
0	0	1	0	10
0	0	1	0	10
0	1	1	0	110
1	0	0	1	1
0	1	0	0	100
0	1	0	0	100
0	0	1	0	10
0	1	0	0	100
0	1	1	0	110
0	0	1	0	10
1	1	1	0	110
0	1	0	0	100
0	1	1	0	110
0	1	0	0	100
0	1	1	0	110
0	1	1	0	110
0	0	1	0	10
0	1	0	0	100
0	0	0	1	1
0	1	0	0	100
1	1	0	0	100
1	1	0	0	100
0	1	0	0	100
0	1	1	0	110
0	1	0	0	100
0	1	0	0	100
0	1	0	0	100
0	0	1	0	10
1	1	0	0	100
0	1	1	0	110
0	1	0	0	100
1	1	0	0	100
0	1	0	0	100
0	1	0	0	100
0	1	1	0	110

0	1	0	0	100
0	1	0	0	100
0	1	0	0	100
0	1	0	0	100
0	1	0	0	100
1	1	0	0	100
0	1	0	0	100
0	1	1	0	110
0	0	1	0	10
0	1	0	0	100
1	1	0	0	100
1	1	0	0	100
0	1	0	0	100
0	1	0	0	100
1	1	0	0	100
1	1	0	0	100
1	1	0	0	100
1	1	0	0	100
0	1	0	0	100
1	1	0	0	100
1	1	0	0	100
0	1	0	0	100
1	1	0	0	100
1	1	0	0	100
1	1	0	0	100
0	1	0	0	100
1	1	0	0	100
1	1	0	0	100
1	1	0	0	100
1	1	0	0	100
0	1	0	0	100
0	0	0	1	1
1	1	0	0	100
0	1	1	0	110
0	1	0	0	100
1	1	0	0	100
0	1	1	0	110
0	1	0	0	100
0	1	1	0	110
0	1	0	0	100
0	1	0	0	100
0	1	0	0	100
0	1	0	0	100
0	1	0	0	100
0	1	1	0	110
0	1	0	0	100
0	1	0	0	100
0	1	0	0	100
1	1	0	0	100
0	1	0	0	100

0	1	0	0	100
0	1	0	1	101
0	1	0	0	100
0	1	0	0	100
0	1	0	0	100
0	1	0	0	100
0	1	0	0	100
0	1	0	0	100
0	1	0	0	100
0	1	0	0	100
0	1	0	0	100
0	1	1	0	110
0	1	0	0	100
0	1	0	0	100
0	0	0	1	1
0	1	0	0	100
0	1	0	0	100
0	1	0	0	100
0	0	0	1	1
0	1	1	0	110
0	0	0	1	1
1	0	1	0	10
1	0	1	0	10
1	1	1	0	110
0	1	0	0	100
0	0	1	0	10
1	1	0	0	100
0	1	0	0	100
1	1	0	0	100
1	1	0	0	100
1	1	0	0	100
1	1	0	0	100
1	1	0	0	100
1	1	0	0	100
0	1	0	0	100
1	1	0	0	100
1	0	1	0	10
1	1	0	0	100
0	1	0	0	100
0	1	0	0	100
1	1	0	0	100
0	0	1	0	10
1	1	0	0	100
1	1	0	0	100
0	1	0	0	100
1	1	0	0	100
1	1	0	0	100
1	1	0	0	100
0	1	0	0	100
0	1	1	0	110

0	1	0	1	101
1	1	0	0	100
1	0	1	0	10
1	1	0	0	100
1	1	0	0	100
1	1	0	0	100
0	1	0	0	100
1	1	0	0	100
1	1	0	1	101
1	1	0	0	100
1	0	1	0	10
0	1	1	0	110
0	0	0	1	1
0	1	0	0	100
0	1	0	0	100
1	0	1	0	10
1	0	0	1	1
0	1	0	1	101
0	1	0	0	100
0	0	1	0	10
0	0	1	0	10
0	1	1	0	110
0	0	1	0	10
0	1	1	0	110
0	1	0	0	100
0	1	0	0	100
0	0	1	0	10
0	1	0	0	100
0	1	0	0	100
0	1	1	0	110
1	1	0	0	100
1	1	1	0	110
0	1	1	0	110
0	1	0	0	100
1	1	1	0	110
1	1	0	0	100
1	1	0	0	100
1	0	1	0	10
1	1	0	0	100
0	1	0	0	100
0	1	1	0	110
0	0	1	0	10
0	1	0	0	100
0	1	0	1	101
0	1	1	0	110
0	0	1	0	10
0	0	1	0	10
0	1	0	0	100
1	0	1	0	10
0	1	0	0	100

1	1	1	0	110
1	1	1	0	110
1	1	0	0	100
0	1	1	0	110
0	1	0	0	100
0	1	0	0	100
0	1	0	0	100
0	1	0	0	100
0	1	1	0	110
0	1	0	0	100
0	1	0	0	100
0	1	0	0	100
0	1	0	0	100
0	1	0	0	100
0	1	0	0	100
0	1	0	0	100
0	1	0	0	100
0	1	0	0	100
0	1	0	0	100
0	1	0	0	100
